# The social circumstances of the maternal experience and its biobehavioral associations, in rhesus macaques (*Macaca mulatta)*

**DOI:** 10.1016/j.anireprosci.2026.108149

**Published:** 2026-02-23

**Authors:** Alexander J. Pritchard, Rosemary A. Blersch, Emily M. Dura, Christina M. Nord, Amy C. Nathman, Jessica J. Vandeleest, Brenda McCowan

**Affiliations:** a Neuroscience and Behavior Unit, California National Primate Research Center, University of California Davis, Davis, CA, USA; b Department of Population Health & Reproduction, School of Veterinary Medicine, University of California Davis, Davis, CA, USA; c Behavioral Management Unit, California National Primate Research Center, University of California Davis, Davis, CA, USA

**Keywords:** Shared motherhood, Allostatic load, Multifactorial, Primates, Breeding season

## Abstract

Motherhood is a physiologically and behaviorally demanding process. We sought to examine how such changes might be expected to alter a mother’s social position within her group and whether there were physiological changes concomitant to these dynamics. We recorded contact-sitting, grooming, huddling, and proximity behavior over two birth and breeding seasons on 120 females across two mixed-sex groups of rhesus macaques (*Macaca mulatta*) at the California National Primate Research Center. We also collected blood samples (from all available females in the first birth season and *n* = 60, thereafter) for blood chemistry and cytokine assays. The reproductive stage of females exhibited a strong influence on the group’s sociality. We observed meaningfully increased connectivity and investment for proximity and contact-sit beginning in late pregnancy continuing after birth, relative to non-mothers, while accounting for past offspring count. Grooming connectivity and investment meaningfully increased during pregnancy, compared to non-mothers, but decreased after the first 50 days postpartum. The number of infants in the group meaningfully increased social associations for all behaviors, except huddling, yet only in the first birth season. Maternal assortment increased, likely due to a higher number of mothers available as social partners; postpartum, however, kin assortativity did not dramatically change. Reproductive status predicted variation in biomarkers whereby pregnant females had meaningfully decreased blood chemistry measures but increased cytokines, relative to non-pregnant females. In summary, maternal social behavior exhibited marked differences across the phases of pregnancy and postpartum that exhibited distinct changes across behaviors, yet these changes in social behavior were not associated with physiological variation beyond that associated to shifts in maternal status.

## 1. Introduction

The maternal experience produces measurable changes in mothers' physical and cognitive functioning, throughout early pregnancy into offspring infancy (Chechko and Nehls, 2025; Paternina-Die et al., 2024; Servin-Barthet et al., 2025). Among many primate taxa these changes do not occur in isolation, rather mothers are embedded in complex social groups that also respond to the presence of infants (Dunayer and Berman, 2017; Fedurek and Lehmann, 2017; Gumert, 2007; Henzi and Barrett, 2002; Suomi, 2005; Tiddi et al., 2010). Thus, understanding the maternal process necessitates consideration of the group condition. This phenomenon is especially prevalent among population that exhibit synchrony in annual cohort births – such as rhesus macaques (*Macaca mulatta*) (Hernández-Pacheco et al., 2016; Oates-O’Brien et al., 2010; Rawlins and Kessler, 1985; Small, 1990; Stavisky et al., 2018).

Motherhood is a recurring, near-universal state for the majority of adult females within stable primate groups. Motherhood, though, is a physiologically demanding process. As such, the duress of pregnancy and early motherhood could be framed as a stressor or characterized by negative affective states (Rezaie-Keikhaie et al., 2020). Indeed, heightened psychosocial stress and low social support have been associated with increased biomarkers of inflammation among pregnant women (Coussons-Read et al., 2007). To manage pregnancy as a stressful experience, human and nonhuman mothers could rely on pre-existing strong stable bonds as a form of social support (Cohen and Wills, 1985; Crockford et al., 2008; Rahmawati et al., 2025; Uchino et al., 1996; Wittig et al., 2008). For instance, primiparous human mothers have been shown to shift their investment to friends, and away from coworkers (Cronenwett, 1985). Similarly in macaques, peripartum mothers have been shown to socially withdraw: generally reducing social approaches, proximity, and allogrooming given, while increasing active withdrawals (Bardi et al., 2003, 2001).

Given the co-occurrence of the maternal experience, females could socially and physiologically benefit from focusing on relationships that emerge through these shared maternal experiences. That is, shared experiences may reinforce or promote new bonds. For example, in humans, parents entering an established school community have been observed to rely on weak bonds and triadic closure to establish network connectivity, while established parents focus on upholding social norms without broadening their networks (Wanat, 2010). Studies in nonhuman primates demonstrate that mothers can, at times, increase their social proximity to others (Altmann, 1980; Brent et al., 2013), a pattern that stands in contrast to reports of postpartum social withdrawal (Bardi et al., 2003, 2001). Such equivocal results indicate the importance of examining multiple behaviors across a multitude of reproductive states while accounting for changes in partner composition and the timing of these changes, relative to that of the group. That is, here we emphasize the importance of simultaneously examining individual maternal experiences across the stages of pregnancy, birth, and infancy, alongside the dynamics of the group.

These maternal experiences are concurrent to changes in the current group composition – i.e., the ratio of mothers to non-mothers. Indeed, there are demonstrated group-level effects for the number of infant births (Dunayer and Berman, 2017; Fedurek and Lehmann, 2017; Gumert, 2007; Henzi and Barrett, 2002; Suomi, 2005; Tiddi et al., 2010). Mothers are expected to experience an even stronger increase in social attention or engagement during periods of low infant bio-availability (Henzi and Barrett, 2002). Furthermore, the biochemical restriction of pregnancy (i.e., birth control) has been shown to alter social engagement (Giraud et al., 2021; Young et al., 2021). Thus, the ratio of mothers to non-mothers is consequential in considering the social dynamics of individual mothers and of the group itself.

Collectively, these dynamics suggest a complex interplay of social and physiological processes that might exert opposing pressures on females. Accordingly, our central aims were twofold: (1) To characterize the biobehavioral change associated with the groups’ synchronized maternal experience. (2) To specifically determine how the shared maternal state reshapes patterns of social connectivity, and whether such shifts buffered or intensified allostatic load. We explored these dynamics in two outdoor-housed, mixed-sex groups of rhesus macaques as a socially complex model system. These findings have the capacity to inform how reproduction alters individual and group social dynamics and individual physiological performance. Furthermore, we gain insight into implications of changes to this system, especially those that can occur in managed free-ranging or captive groups.

### 1.1. Hypotheses and predictions

Broadly, we hypothesized that the maternal experience would be reflected through both behavioral and physiological indicators. While this hypothesis does not challenge established theory, our contribution lies in simultaneously examining multiple social and physiological measures, along with their concurrent dynamics. We address two primary questions: How do pregnancy and motherhood alter a mother’s social position within her group, and how persistent are these behavioral changes? What are the nature of the physiological changes concomitant to these social dynamics?

Behaviorally, we expected that newborns may accrue increased social attention that may or may not be desirable to mothers (Dunayer and Berman, 2017; Henzi and Barrett, 2002). Under this presupposition, the social connectivity of mothers with conspecifics might increase. Alternatively, evidence suggests that mothers might concentrate their bonds or rely on fortifying weak bonds (Crockford et al., 2008; Cronenwett, 1985; Wanat, 2010; Wittig et al., 2008). Under this presupposition, overall social investment or connectivity might be focused on particular individuals. Prior work found little support that maternal status affects grooming behavior, while findings for social proximity were mixed (Brent et al., 2013). There are, however, likely distinct phases under which different types of distinct changes might manifest. For example, early infancy might be defined by fortifying strong bonds with a social preference towards close friends and family – either as a response to a stressor or due to limited energy for socializing. While later infancy into juvenility might be defined by maternal investment in diversifying to and strengthening weaker bonds.

Physiologically, individuals that do not have satisficing social support are expected to exhibit evidence of allostatic load (i.e., physiological ‘wear and tear’ [McEwen and Stellar, 1993; McEwen and Wingfield, 2003; Romero and Wingfield, 2015]), relative to individuals with satisficing support. This presents a challenge because at least three states must be conceptually differentiated: 1) individuals in homeostasis (i.e., without elevated biomarkers), 2) individuals experiencing allostatic load (i.e., with elevated biomarkers), and 3) individuals in homeostasis through social support (i.e., with social buffering that keeps biomarkers in homeostasis, despite stressors). Distinguishing between individuals in homeostasis is more challenging than distinguishing between animals in homeostasis and those entering a state of allostatic load. To complicate this dynamic, allostatic load and blood chemistry indicators can vary across pregnancy (Bakker et al., 2023; Chandra et al., 2012; Coussons-Read et al., 2007; Ibáñez-Contreras et al., 2013; Li et al., 2020; Orsi et al., 2006; Wu et al., 2015). To partially mitigate these dynamics, we elected to examine allostatic load as a phenomenon that can emerge via multiple dimensions (King et al., 2019; Lueth, 2022); this approach accommodates, but does not constrain, allostatic load as a unidimensional index. That is, allostatic load may not be unidirectional, with some individuals exhibiting predispositions towards deleterious outcomes in particular biological systems (e.g., cardiac versus liver function). This rationale was consistent with our study populations as these were healthy animals unlikely to enter states of allostatic overload characterized by severe multiple biomarker decline, which would necessitate medical intervention. Rather, any physiological shifts were expected to reflect changes in chronic or environmental stress (i.e., allostatic load). We focused on blood chemistry and three cytokine measures that have been studied in rhesus macaques and implicated in welfare or variation in allostatic load (Capitanio et al., 2023; Coussons-Read et al., 2007; King et al., 2019; Li et al., 2020; Lueth, 2022; Orsi et al., 2006; Vandeleest et al., 2016).

## 2. Materials and methods

**Fig. 1.**
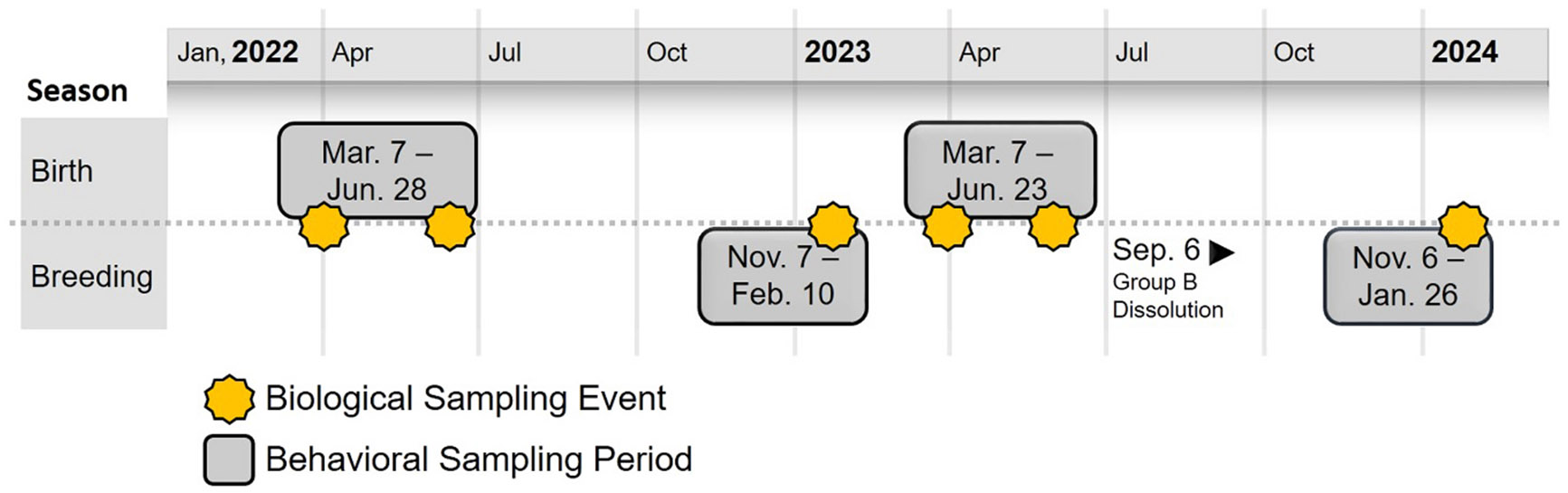
Study timeline. Note that the dates of behavioral collection were similar between the two groups, though group B was dissolved before the last collection period.

We conducted this study at the California National Primate Research Center (CNPRC), from March, 2022, to January, 2024. The study took place across two groups of rhesus macaques with four observation periods composed of two consecutive birth seasons and two consecutive breeding seasons (Fig. 1). We label these study periods by season and year combination: BiY1, BrY1, BiY2, and BrY2 where Birth and Breeding are abbreviated as Bi and Br, while Year One and Year Two are abbreviated as Y1 and Y2, respectively. Although both groups were perceived to be stable at the onset of the study, Group B experienced a social overthrow in September of 2023 resulting in a disbandment of the majority of group members – thus, only one group was studied for BrY2. Project design and reproductive management interventions were approved by an Institutional Animal Care and Use Committee and employed standard colony practices.

### 2.1. Study animals

Across the entire study period, there were 162 animals in group A and 184 in group B. Animals were housed in their home half-acre outdoor enclosures with *ad libitum* access to water and monkey chow, plus minimums of twice-daily seed provisioning, and weekly or semiweekly produce. We collected behavioral data on all animals over the age of three, which included 55 F: 30 M in group A and 65 F: 33 M in B. Biomarker data in BiY1 were collected from all females over the age of three, at that time. Thereafter, we focused on 30 females per group (*n* = 60). In BrY1, ten of the subjects were on birth control per group. In BiY2, 38 females entered their third trimester during observations. After birth, six females per group had their infant relocated to lactating foster mothers in other housing units. All reproductive management interventions were approved by an Institutional Animal Care and Use Committee and employed standard colony practices.

### 2.2. Behavioral data

Behavioral data were collected via scan sampling for affiliative behaviors and event sampling for agonistic behaviors. The latter included displacements, which were used to calculate social dominance hierarchies, detailed below. Affiliative data included all unique dyads engaging in grooming, contact-sitting, huddling, or were in proximity to within one female arm’s length. Whole group scans were conducted every 20 min and observations occur from 0900 to 1200 h and 1300–1600 h. Observations were alternated between groups by calendar week. Two observers observed a single enclosure during the same week, one collecting agonistic observations and another recording affiliative behaviors. Observers achieved an interobserver reliability of Krippensdorf’s *α* ≥ 0.85 prior to observations. Due to longer observation periods during the birth period, we collected data two days a week, while during the breeding period we collected data four days a week.

### 2.3. Biomarker samples

We collected biological samples during whole group sampling events. Animals were sampled in their home enclosure and were anesthetized with ketamine (10 mg/kg) for general health checks, including femoral blood draws. The two groups were sampled on different days. We had two sampling events for each of the birth seasons (March/April and June), and one sample event for each of the breeding seasons (January). The average numbers of samples for the 30 female subjects sampled across the project were 5.42 ± 0.62*sd*. We also had two samples for 37 of the 40 additional females sampled in BiY1, with a single sample for the remaining three subjects. In total, we had 402 biological serum samples that were stored at −80°C from the time of collection until assaying for biomarkers.

### 2.4. Effect of pregnancies and births on social behavior

To assess the behavioral consequences of pregnancy and birth, we modelled counts of social behavior. We, initially, included all adult subjects. We extracted all counts of proximity, contact-sit, huddling, and grooming. To examine general social behavior, we used undirected data for each day of observation to capture nuances of progressive change. We populated all zeroes for females who were physically present in the enclosure, but did not express or receive a behavior on a given observation day. After tallying behavioral counts, we removed males from subsequent analyses such that males were included as social partners but only females were examined as study subjects.

The timing of infant births varied by mother and, thus, we labelled females relative to their infant’s birth: second trimester (> 50 days until birth), third trimester (≤ 50 days until birth), early infancy (≤ 50 days postpartum), later infancy (> 50 days postpartum), as donors (infant relocation to foster mother), or as non-mothers (no infant). We calculated infant count within each group as a running tally of births prior to each date, not counting fostered infants. In the first study birth season, the median ± Interquartile Range (IQR) of lifetime births was 1 ± 5 IQR among all female subjects and 4 ± 4 IQR when excluding nulliparous females.

For each female, we calculated social dominance rank and their total lifetime births. For social dominance rank, we used percolation and conductance in the *Perc* R package (Fujii et al., 2016) on all occurrences of event sampled displacement interactions, with a max length of 4 and the refine function. The best simulated ordinal ranks were converted to the percentages of animals outranked. For lifetime births, we tallied the number of offspring prior to project start (used for the first birth season), plus the number of offspring born in the first birth season (for the second birth season).

Climatic changes are expected to co-occur with the progression of pregnancy and infant development, as reproductive behavior can vary by season in this population (Pritchard et al., 2025a). To disentangle these effects, we included temperature as a covariate. Temperature was selected as generally representative of climatic patterns as high relative humidity and rainfall were primarily observed seasonally, during the winter. We obtained daily temperatures collected at a nearby weather station using the Russell Ranch and Campbell Tract sensors at 2 m (UCD/NOAA Climate Station: USC00042294 [Atmospheric Science Program, Land Air and Water Resources, University of California Davis, 2025; Menne et al., 2012]). For each observation day, we extracted the maximum temperature between 9:00 and 15:00.

### 2.5. Effect of pregnancies and births on local networks

We sought to assess local network changes among females experiencing motherhood. We used three measures: assortativity, Jaccard similarity, and transitivity. To determine whether females changed who they associated with based on properties of kinship or shared motherhood, we quantified differences between assortativity pre- and postpartum. Assortativity refers to a nonrandom connectivity with similar (homophily) or dissimilar (heterophily) partners on a trait of interest (Farine, 2014; Newman, 2002). To determine whether mothers have (dis)similar connections and close transitive connections in the breeding season relative to the preceding birth season, we calculated Jaccard and transitivity indices. Jaccard indices provides a measure of (dis)similarity giving insight into whether females formed new bonds or retained their original bonds. Transitivity across seasons provides insight into whether females closed bonds between friends-of-friends.

We created ego networks using the same birth bins, as defined above: second trimester, third trimester, first 50 days, and infancy. For each behavior, we compared how assortativity in each period of infancy differed from each of the periods of pregnancy (i.e., two-by-two comparison). In line with our expectations, postpartum mothers might be expected to, either, focus on kin or on non-kin. If females are focused on non-kin, we might expect them to invest in females who are also new mothers. These analyses are important in conjunction with the prior analysis because these assortativity indices examine *who* females are investing in.

To examine whether connections during early infancy (i.e., when females are undergoing shared motherhood) were maintained during the following breeding season, we pooled both trimester bins into a pregnancy network and pooled both the first 50 and infancy periods bins into a birth season bin. We then created a new network for each of the females present during the breeding season. For Jaccard indices, these networks were limited to direct connections. For transitivity, these networks were limited to direct connections and their direct connections during the birth season.

### 2.6. Biomarkers

#### 2.6.1. Biomarker assays

Cytokines were quantified using the MILLIPLEX^®^ Non-Human Primate Cytokine/Chemokine/Growth Factor Panel A (PRCYTA-40K) by MilliporeSigma^®^ (Luo et al., 2021). We assayed three biomarkers using this kit: Interleukin-6 (IL-6), Interleukin-10 (IL-10), and Tumor Necrosis Factor (TNF, also known as TNF-α [Grimstad, 2016]). Assays utilized Luminex 200 System with xPONENT 3.1 (Luminex, Austin, TX, USA). Samples were run in duplicate. Though the assay has been validated for use in rhesus macaques, many of our detectable samples had high intraassay coefficients of variation (CV). Among our detectable samples, 4 % of IL-6, 14 % of IL-10, and 40 % of TNF exceeded a CV of 20 %.

Contingent on the cytokine, numerous samples were out of the detectable threshold (<2.44 for IL-6 and IL-10; <4.88 for TNF). If both sample duplicates were detectable, then we calculated the mean between them. If either sample duplicate was undetectable or both read below the detectability threshold, then we set the value at 0.00. This creates a simplifying assumption that all below-threshold reads were equivalent to zero. Only one subject was below threshold for all cytokines. This point is relevant because all other subjects had true detectable values to assign factor scores. We also elected to recode TNF to a dichotomous variable, where all samples below the detectable threshold were assigned a zero, while all detectable values were assigned a one. Model fit was similar before and after recoding. This decision was, in part, due to the number of undetectable TNF samples and due to the high CVs for detectable samples.

Blood chemistry tests were run on a Beckman Coulter AU480 analyzer by the CNPRC Clinical laboratory using their commercial reagents for: Sodium (NA), Potassium (K), Chloride (CL), bicarbonate (CO2), Anion Gap (AGAP), Phosphorus (PHOS), Calcium (CA), Blood Urea Nitrogen (BUN), Creatinine (CREA), Glucose (GLU), Total Protein (TP), Albumin (ALB), Alanine Aminotransferase (ALT), Aspartate Aminotransferase (AST), Creatine Kinase (CPK), Alkaline Phosphatase (ALKP), γ-glutamyl transferase (GGT), Lactate Dehydrogenase (LDH), Cholesterol (CHOL), Triglyceride (TRIG), Total or Direct Bilirubin (TBIL and DBIL, respectively), high sensitivity C-reactive protein (CRP), and Albumin (ALB). Quality control was run each morning before any sample processing. Samples were run in singlicate unless samples were above established critical ranges, in which case a replicate was run to confirm the reading.

#### 2.6.2. Factor model

We conducted an exploratory factor analysis to simplify the 23 blood chemistry and three cytokine biomarkers into fewer variables of interest. We utilized several pre- and post-screening methods to assess and improve factor model validity (Costello and Osborne, 2005; Morton and Altschul, 2019). Prior to constructing our factor models, we assessed Kaiser-Meyer-Olkin Measures of Sampling Adequacy (MSA) with a threshold of ≥ 0.50 for each biomarker, utilizing KMO in the *psych* package (Revelle, 2023). We used automated methods to select the number of factors (Morton and Altschul, 2019), utilizing the *psych* package functions (nfactors, fa. parallel) (Revelle, 2023). For our factor model, we confirmed that all biomarkers had a communality ≥ 0.40. We repeated each of these steps, dropping any violating measures, until the model fulfilled all of these criteria. We report dropped biomarkers and the reason for exclusion in Supplementary Text 1. We used weighted least squares factor method to accommodate this dichotomous recoding. The overall MSA was 0.76. The resulting model had four factors (Table 1) that explained 61 % of the cumulative variation. Root mean square of the residuals, with degrees of freedom corrected, was 0.04 and below the acceptable threshold of < 0.08. Each of the four factors broadly represented, respectively, markers of blood protein, tissue damage, inflammation, and liver function. Regression factor scores were extracted for each subject and then increased by the minimum value, plus 0.01, so that factor scores were positive and non-zero.

### 2.7. Statistical analyses

#### 2.7.1. Models for effect of pregnancies and births on social behavior

We fit mixed effects Bayesian Regression models using Stan with *brms* in R (Bürkner, 2018, 2017). We ran models using weakly-informative (normal [0,1]) priors for our fixed effects, 4 chains, 3000 iterations with a warmup of 1500, and a thin of 2 – resulting in 3000 draws. We had four models, one for each behavior, with count as a response variable. We used hurdle negative binomial distributions, which facilitates quantifying effects of both connectivity (via the hurdle) and investment (via non-zero counts).Predictors included: rank, social group, count of lifetime offspring, maximum temperature, birth status, infant counts per group, and study period. Preliminary models supported the inclusion of an interaction between infant count and period. This effect was not anticipated, but more accurately reflected the real social dynamics in both groups. We included random effects for subject identifier. The hurdle portion of the model replicated the same model structure. Our selected models fit the data well, as assessed via visual inspections of posterior predictive checks (Supplementary Figure 1) and pairs plots (Supplementary Figure 2) from *brms*, as well as $\hat{R}$ and effective sample size estimates (near 1.0 and exceeding 1000, respectively; Supplementary Tables 1-4). Meaningful associations were determined as differing from zero at the 95 % credible interval.

Our models on social behavior explained between 33 % and 16 % of behavioral variance with fixed and random effects, and 24–12 % with fixed effects alone (Supplementary Table 5). The model that explained the most variance was the proximity model, followed by huddling, grooming, and then contact-sit.

#### 2.7.2. Models for effect of pregnancies and births on biomarkers

We used log-linked gamma distributions for our factor score regression models, with the exception of a normal (Gaussian) distribution for factor one. We ran models using weakly-informative (normal [0,1]) priors for our fixed effects, 4 chains, 3500 iterations with a warmup of 1000, and a thin of 2 – resulting in 5000 draws. Predictors included: rank, social group, lifetime offspring, season, year, birth status, and behavioral rates of unique dyadic associations for proximity, grooming, and contact-sit, per day. We also included whether subjects were on birth control during the breeding season, which was true in year two for 20 of our subjects. We included random effects for subject identifier. Our selected models fit the data well, as assessed via visual inspections of posterior predictive checks (Supplementary Figure 3) and pairs plots (Supplementary Figure 4), as well as $\hat{R}$ and effective sample size estimates (near 1.0 and exceeding 1000, respectively; Supplementary Tables 6-9). Meaningful associations were determined as differing from zero at the 95 % credible interval (CI).

Our models explained between 78 % and 42 % of biomarker factor score variance with fixed and random effects, and 54–6 % with fixed effects alone (Supplementary Table 10). The model that explained the most variance was the factor four model, followed by factors one, and then factor three had the lowest conditional R^2^, while factor two had the lowest marginal R^2^.

## 3. Results

### 3.1. Effect of pregnancies and births on social behavior

#### 3.1.1. Maternal dynamics

**Fig. 2.**
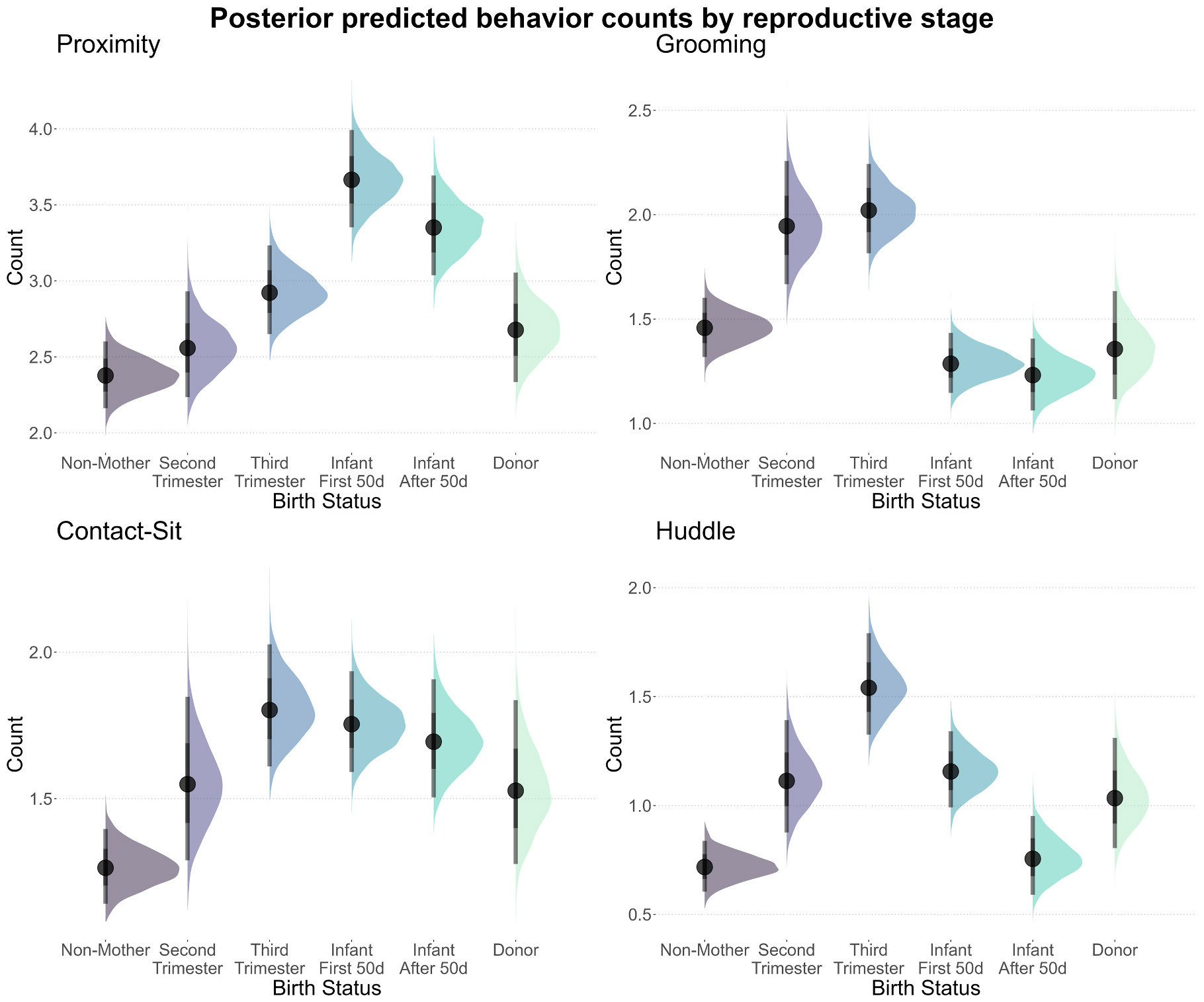
Model posterior draws shown using half-eye and interval density plots for undirected behavioral counts (y-axes) across the categories of reproductive status (x-axis). Behavior are, clockwise from top-left: proximity, grooming, huddling, and contact-sit.

We aimed to quantify the general contributions of pregnancy, birth, and infant availability to social behavior. Relative to non-mothers, the birth of an infant increased social connectivity and investment for proximity (Connectivity: −1.51, 95 % CI = −2.02_Lower_ to −1.03_Upper_; Investment: 0.39 estimate, 95 % CI = 0.30_Lower_ to 0.47_Upper_) as well as contact-sit (Connectivity: −0.67, 95 % CI = −0.92_Lower_ to −0.44_Upper_; Investment: 0.29 estimate, 95 % CI = 0.15_Lower_ to 0.41_Upper_) (Fig. 2; Supplementary Tables 1-4). Connectivity estimates influence the probability of attaining a zero, such that negative values indicate a higher probability of connectivity and positive values indicate a lack of connectivity. These effects were also found for contact-sit in the third trimester, relative to non-mothers (Connectivity: −0.79, 95 % CI = −1.05_Lower_ to −0.51_Upper_; Investment: 0.30 estimate, 95 % CI = 0.15_Lower_ to 0.44_Upper_), and in the second trimester for proximity connectivity (Connectivity: −1.06, 95 % CI = −1.75_Lower_ to −0.43_Upper_), but not investment. For grooming, however, mothers differed from non-mothers with increased grooming connectivity and investment during the second (Connectivity: −0.72, 95 % CI = −1.20_Lower_ to –0.22_Upper_; Investment: 0.21 estimate, 95 % CI = 0.03_Lower_ to 0.38_Upper_) and third trimesters of pregnancy (Connectivity: −0.74, 95 % CI = −1.05_Lower_ to −0.45_Upper_; Investment: 0.26 estimate, 95 % CI = 0.14_Lower_ to 0.37_Upper_). Relative to non-mothers, grooming investment decreased after birth (−0.12 estimate, 95 % CI = −0.24_Lower_ to −0.01_Upper_) and continued through the first 50 days of infancy (−0.16 estimate, 95 % CI = −0.31_Lower_ to −0.02_Upper_), but connectivity was unchanged. For huddling, connectivity increased from the second trimester (−0.71 estimate, 95 % CI = −1.14_Lower_ to −0.31_Upper_) through the first 50 days of infancy (−0.62 estimate, 95 % CI = −0.87_Lower_ to −0.38_Upper_), yet not later infancy; while investment increased from the third trimester (0.48 estimate, 95 % CI = 0.32_Lower_ to 0.65_Upper_) through to late infancy (0.33 estimate, 95 % CI = 0.11_Lower_ to 0.55_Upper_), all relative to non-mothers.

Donor mothers did not differ from non-mothers with connectivity and investment for proximity and grooming. For contact-sit, however, donors had uncertain evidence (i.e., CIs approximating, but not crossing, zero; 0.26 estimate, 95 % CI = 0.0004_Lower_ to 0.52_Upper_) for higher investment, yet not connectivity – perhaps reflective of declining trends during pregnancy. For huddle, donors had higher connectivity (−0.57 estimate, 95 % CI = −1.00_Lower_ to −0.14_Upper_), yet not investment, relative to non-mothers.

**Fig. 3.**
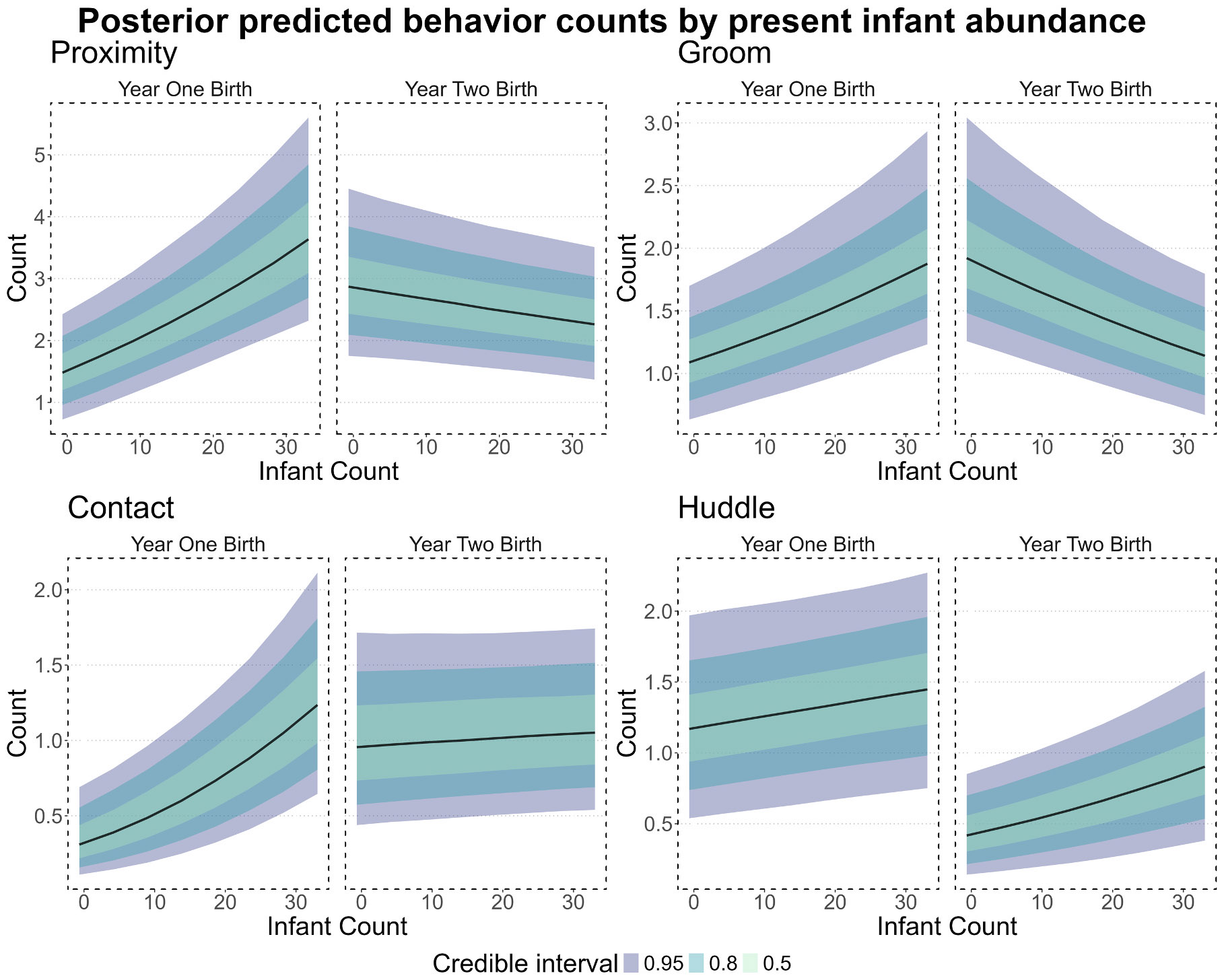
Posterior estimates for counts of each behavior. Behaviors are, clockwise from top-left: proximity, grooming, huddling, and contact-sit. Scaled infant count (x-axis) as a predictor of behavior count (y-axis) in each of the two birth seasons (panels). Note the change in association between seasons.

We sought to explicitly disentangle the maternal effect from general trends of female interest in infants. During the first study season, proximity, grooming, and contact-sit all increased in connectivity and investment as the number of infants in the group increased (Fig. 3; see Supplementary Tables 1-4 for estimates and CIs). This association was not present with certitude for huddling behaviors. In the second observation season, however, this effect was reversed for proximity and grooming (more available infants decreased investment in these behaviors) and was nullified for contact-sit. This distinction between the seasons is important to emphasize as Y2 was defined by having a subset of females on birth control and as donors. Finally, past reproductive activity (offspring count) was associated with higher connectivity and investment for proximity (Connectivity: −0.55, 95 % CI = −0.96_Lower_ to −0.14_Upper_; Investment: 0.15 estimate, 95 % CI = 0.05_Lower_ to 0.26_Upper_), grooming (Connectivity: −0.38, 95 % CI = −0.64_Lower_ to −0.12_Upper_; Investment: 0.15 estimate, 95 % CI = 0.04_Lower_ to 0.27_Upper_), and contact-sit (Connectivity: −0.26, 95 % CI = −0.49_Lower_ to −0.03_Upper_; Investment: 0.21 estimate, 95 % CI = 0.09_Lower_ to 0.34_Upper_); but not huddling (Supplementary Tables 1-4). As offspring count and subject age were highly correlated (*r* = 0.95), we cannot wholly differentiate whether offspring count or female age is the relevant driver of these associations. Indeed, whole model predicted estimates with subject age substituted for the number of offspring were highly correlated with our selected model’s estimates (*r* > 0.997).

#### 3.1.2. Socio-environmental dynamics

Although not of primary focus, we found meaningful associations in several socio-environmental variables. Social dominance rank was positively associated with connectivity and investment for proximity (Connectivity: −0.78, 95 % CI = −1.20_Lower_ to −0.39_Upper_; Investment: 0.32 estimate, 95 % CI = 0.21_Lower_ to 0.43_Upper_) and grooming (Connectivity: −0.60, 95 % CI = −0.87_Lower_ to −0.35_Upper_; Investment: 0.41 estimate, 95 % CI = 0.28_Lower_ to 0.53_Upper_), but only with connectivity for contact-sit (Connectivity: −0.29, 95 % CI = −0.54_Lower_ to −0.05_Upper_). Maximum temperature was positively associated with connectivity and investment for proximity (Connectivity: −0.43, 95 % CI = −0.80_Lower_ to −0.08_Upper_; Investment: 0.20 estimate, 95 % CI = 0.13_Lower_ to 0.27_Upper_) and grooming (Connectivity: −0.53, 95 % CI = −0.75_Lower_ to −0.30_Upper_; Investment: 0.11 estimate, 95 % CI = 0.01_Lower_ to 0.21_Upper_), but not with contact-sit. Huddling was negatively associated with maximum daily temperature (Connectivity: 1.26, 95 % CI = 1.04_Lower_ to 1.49_Upper_; Investment: −0.67 estimate, 95 % CI = −0.81_Lower_ to −0.54_Upper_).

### 3.2. Effect of pregnancies and births on local networks

**Fig. 4.**
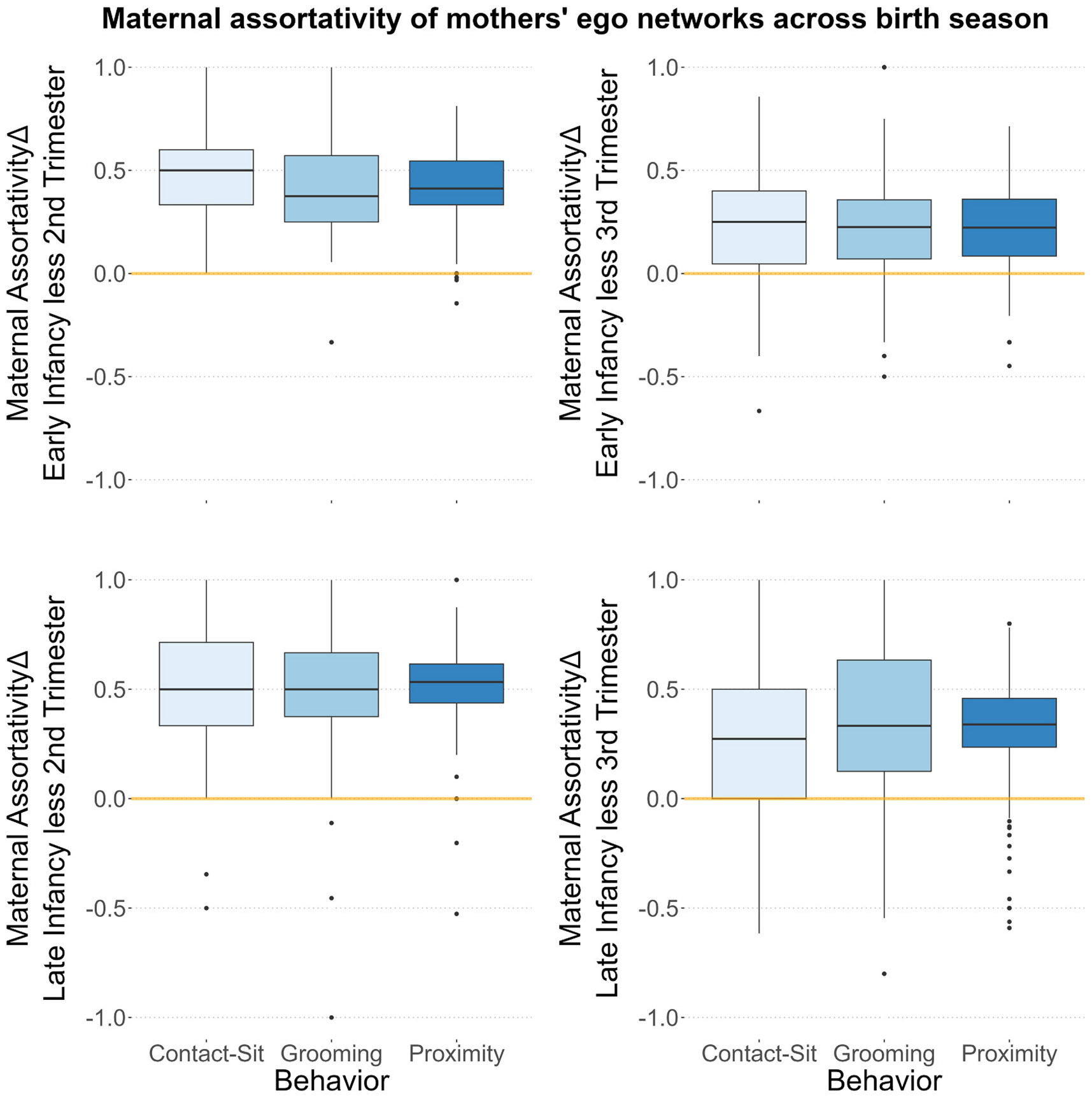
Boxplots of changes to motherhood assortativity with early (top row) or late infancy (bottom row) minus assortativity indices from the second (left column) or third trimester (right column) for three behaviors (x-axis and fill color). Note that equivalence between two periods is emphasized by 0 at the y-intercept.

We sought to determine whether shared maternal experiences promoted social bonds and diversification of connections. In line with this logic, postpartum mothers diversified their bonds towards other, current mothers (Fig. 4), especially for comparisons with the second trimester. Postpartum mothers, however, showed no evidence of diversifying their bonds beyond kin, with consistent measures of kin assortativity (Supplementary Figure 5).

#### 3.2.1. Parity, assortativity, and transitivity

Primiparous females generally had comparable patterns in assortativity and similarity as multiparous females. Maternal assortativity was higher in Y1 among primiparous females, but examination of the birth dates indicated that primiparous females in Y1 gave birth later in the season, when more mothers would be present. Nearly all females had higher transitivity (logical due to network density) during the breeding season, relative to during pregnancy or infancy. Relative to multiparous females, primiparous females showed similar patterns in transitivity during the breeding season, compared to pregnancy and infancy (Supplementary Figure 7).

#### 3.2.2. Network similarity

**Fig. 5.**
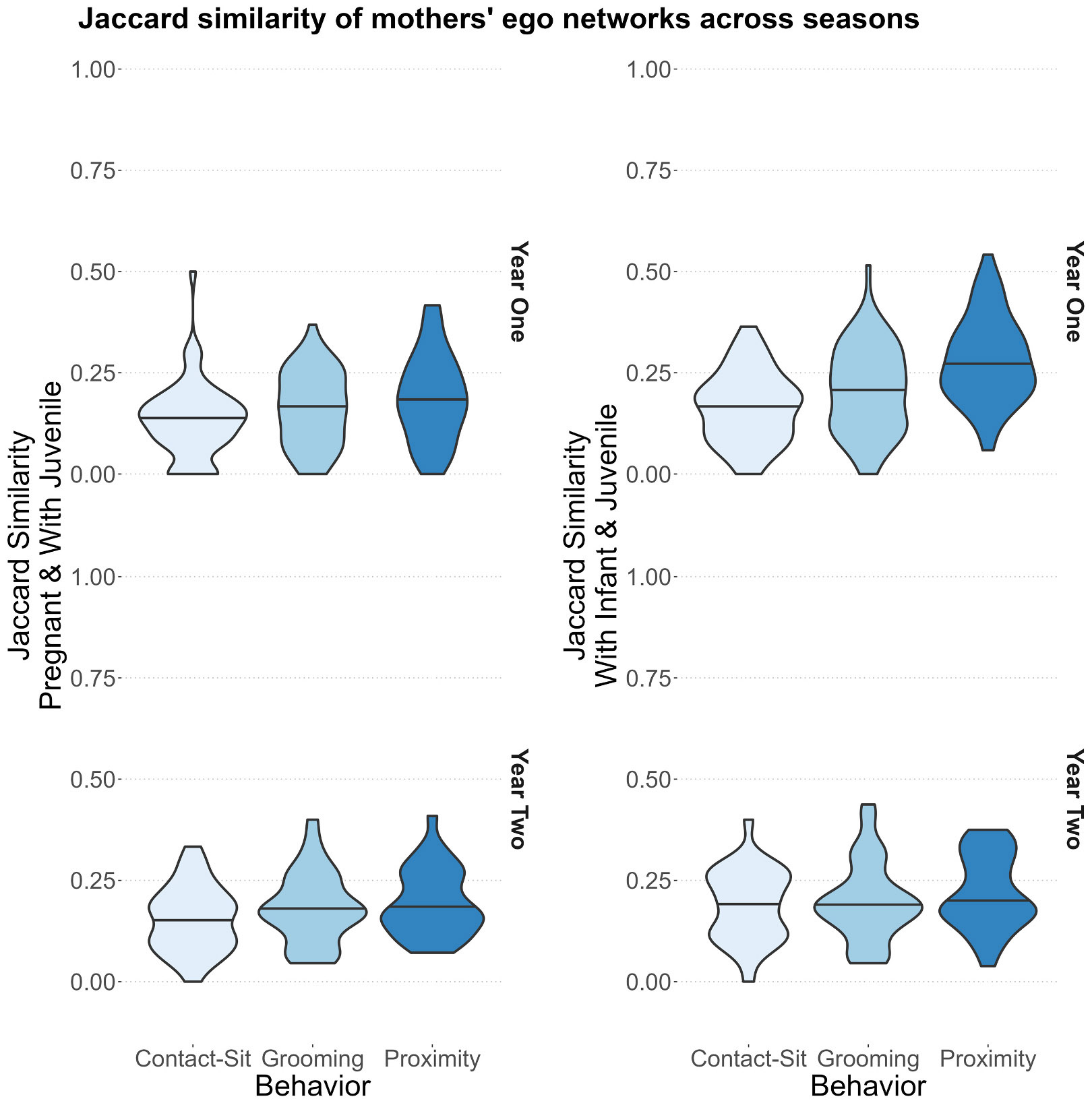
Violin plots of Jaccard similarity indices (y-axes) for three of the behaviors (x-axes; fill color). Plots are organized for year one (top row) or two (bottom row) and compare breeding season associations to associations in the preceding pregnancy (left column) or early infancy (right column).

Maternal and kin assortativity existed despite social turnover: Jaccard indices indicated that all breeding season networks only shared a minority of connections, relative to the periods of pregnancy. The mean similarity indices across all behaviors and comparisons was 0.15, with a maximum of 0.23 (huddling between early infancy and the third trimester) and a minimum of 0.08 (grooming between late infancy and the second trimester) (Supplementary Figure 6). The second trimester had lower similarity to both birth periods (mean = 0.11), relative to the third trimester networks (mean = 0.19). Jaccard indices for pre- and postpartum associations compared to breeding season associations indicated a higher number of shared proximity associations between postpartum and breeding season networks, relative to prepartum comparisons (Fig. 5), yet the size of this effect was isolated to the first study year and small (i.e., the median increase was within the range of variation for comparisons).

### 3.3. Variation in biomarker expression

We aimed to examine how biomarker expression changed as a function of reproductive status, social circumstances, and time. Median blood chemistry values were similar to published norms, though CO^2^ values were high (Bakker et al., 2023; Chen et al., 2009; Koo et al., 2019; Shah et al., 2022). Detectable cytokine values were similar to published values from healthy animals (Duan et al., 2025; Vandeleest et al., 2025, 2016). Our biomarkers were simplified into four factors which represented, broadly, markers of blood protein, tissue damage, inflammation, and liver function.

**Fig. 6.**
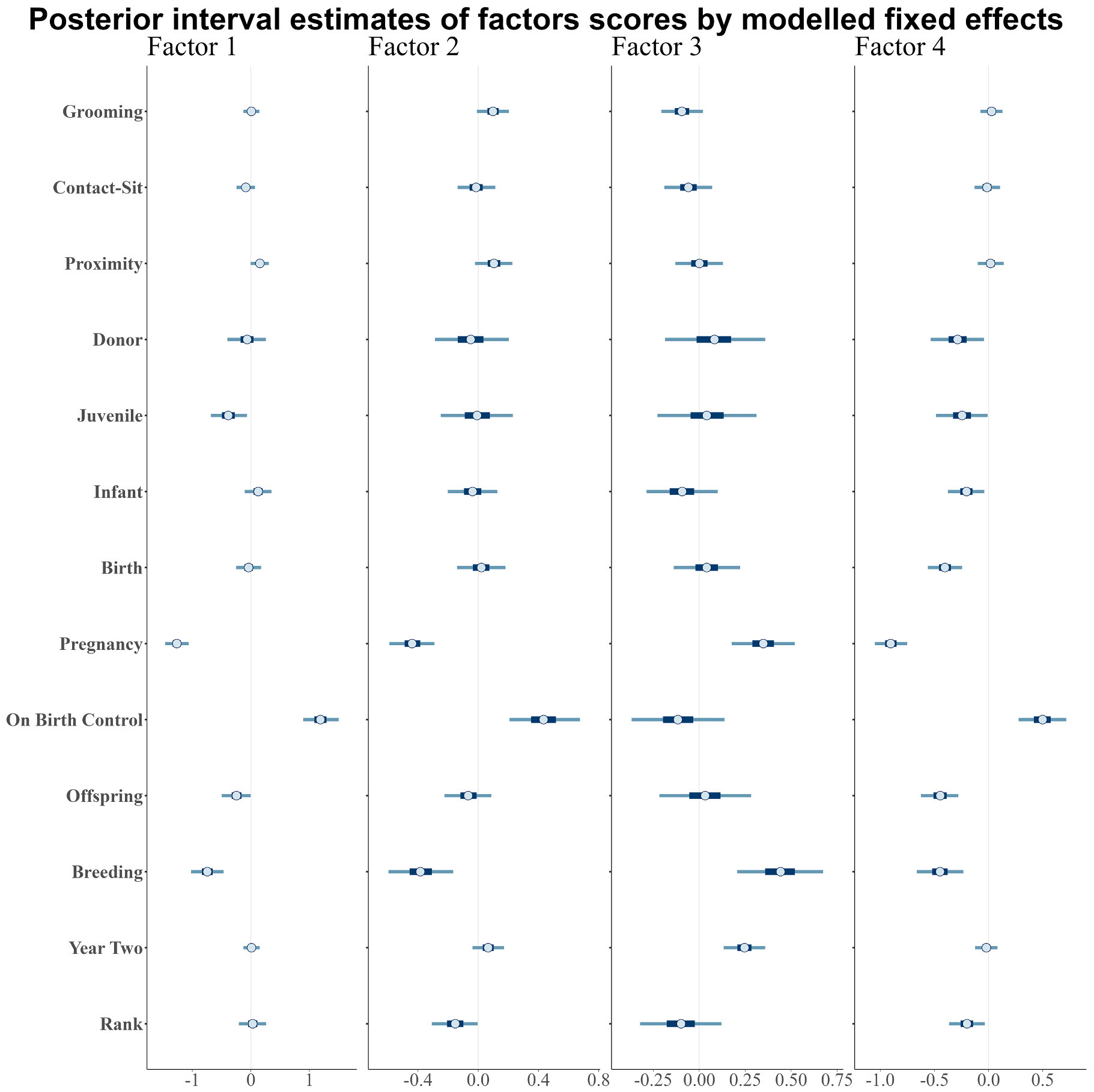
Posterior estimates for the univariate biomarker models. Points show the median estimate, with inner intervals extending to the 50 % credible interval, and outer intervals to the 95 % credible interval. Modelled fixed effect variables are on the y-axis.

Reproductive status was associated with our biomarker factors (Fig. 6; Supplementary Tables 5-8). Relative to non-mothers, pregnant females had credibly different scores for all four factors, with lower scores for factors one (−1.26 estimate, 95 % CI = −1.46_Lower_ to −1.06_Upper_), two (−0.44 estimate, 95 % CI =−0.59_Lower_ to −0.29_Upper_), and four (−0.90 estimate, 95 % CI = −1.05_Lower_ to −0.75_Upper_), but higher scores for factor three (0.35 estimate, 95 % CI = 0.18_Lower_ to 0.52_Upper_). For factor one, having a juvenile (−0.38 estimate, 95 % CI = −0.68_Lower_ to −0.06_Upper_) and the number of offspring (−0.25 estimate, 95 % CI = −0.50_Lower_ to −0.002_Upper_) both decreased scores, though the offspring effect was uncertain. For factor four, these differences continued into the bin that included birth (−0.40 estimate, 95 % CI = −0.56_Lower_ to −0.24_Upper_). Yet, factor four also had smaller and uncertain differences for donor (−0.29 estimate, 95 % CI = −0.53_Lower_ to −0.04_Upper_), juvenile (−0.24 estimate, 95 % CI = −0.49_Lower_ to −0.01_Upper_), and infant mothers (−0.21 estimate, 95 % CI = −0.38_Lower_ to −0.04_Upper_) relative to non-mothers. Across all factors, the breeding season credibly differed from birth season with the same directionality as pregnancy status. Additionally, relative to non-mothers, females on birth control (which was only the case for a subset of females during BrY2) had higher blood chemistry scores (factor one: 1.19 estimate, 95 % CI = 0.89_Lower_ to 1.50_Upper_; factor two: 0.44 estimate, 95 % CI = 0.21_Lower_ to 0.68_Upper_; factor four: 0.50 estimate, 95 % CI = 0.28_Lower_ to 0.72_Upper_), but there was no difference to factor three (cytokine) scores. Increased cumulative offspring (−0.45 estimate, 95 % CI = −0.62_Lower_ to −0.28_Upper_) and social dominance rank (−0.20 estimate, 95 % CI = −0.39_Lower_ to −0.03_Upper_) were both negatively associated with scores on factor four. None of the behavioral rates during the relevant bins were associated with biomarker factor scores.

Due to the inclusion of cytokine samples with high intraassay CVs, we re-ran the factor three model and omitted samples (N = 20) that had high (>20 %) CVs for, either, IL-6 or IL-10. Model output was similar between the model with and without the omitted samples (Supplementary Figure 8).

## 4. Discussion

### 4.1. Effect of pregnancies and births on social behavior

Maternal dynamics exhibited a strong influence on the mothers themselves, and to the social circumstances of their wider group. Social differences were also associated with infant availability, environmental changes, and established social power dynamics (i.e., rank). The maternal effects showed variance relative to the timing of their infants’ birth. Additionally, these effects were not consistent across behaviors, with more passive social processes increasing just before or after birth, which is partially supported by prior work (Brent et al., 2013). Grooming, however, was elevated during pregnancy, yet was lower – at least for investment – after birth relative to non-mothers. The group responded to the bioavailability of infants in line with prior work (Dunayer and Berman, 2017; Fedurek and Lehmann, 2017; Gumert, 2007; Henzi and Barrett, 2002; Suomi, 2005; Tiddi et al., 2010). The gross number of infants increased social engagement. Yet this effect was only clear in our first birth season and was nullified, if not reversed, in our second study season.

During our second study season we had administration of birth control and fostering. This is relevant to the gross bioavailability of infants, but also because the donor subject group provides insight into the relative timing of these social changes. The gross number of infants did not differ between the seasons, but the ratio of potential-to-realized mothers did change. The y-intercepts for contact-sit, proximity, and grooming were higher at the start of the birth season – in support of expectations (Henzi and Barrett, 2002). These behaviors, however, did not increase or decreased as the number of infants increased.

The behavior of the donor subjects demonstrate that changes in grooming – elevated during pregnancy but decreased during infancy – were not wholly attributable to infant presence. Rather, there is likely a physiological or socio-biological drive towards heightened grooming that ceases postpartum. Similarly, the donor group provides evidence that proximity increases solely due to the presence of the infant, as proximity behavior for donors remained similar to that of the third trimester while it was heightened for mothers with infants. This latter observation shows concordance with observations by Altmann (1980) for yellow baboons (*Papio cynocephalus*) and with the findings of Brent et al. (2013) for one of their two groups, but contrasts with observations of decreased maternal social proximity near birth (Bardi et al., 2003). Unlike the work by Brent et al. (2013), however, our findings suggest that the maternal experience *is* of broad consequence to the social dynamics of the group. This distinction we attribute to our focus on daily sociality rather than broad structural changes.

### 4.2. Effect of pregnancies and births on local networks

We examined whether females biased their associations to particular classes of individuals after birth: kin and concurrent mothers. Mothers retained the same kin biases that they held before birth despite a small number of consistent bonds between these periods (~15 % Jaccard similarity). Prior work reported comparable similarity ranges for high-ranking males (Pritchard et al., 2024), suggesting that partner similarities did not deviate markedly across birth periods relative to general social variation. Furthermore, maternal kinship biases are a strong social norm in rhesus macaques (Kapsalis and Berman, 1996; Pritchard et al., 2025b), a pattern that the maternal experience did not markedly alter. This finding was evident here even through the lack of directional change in kin assortativity.

Despite the lack of change in kin assortativity, mothers exhibited a shift towards associating with other concurrent mothers across all of our affiliative behaviors, except huddling. This maternal homophily was difficult to contextualize, because the availability of mothers was concurrently increasing. Given the fewer offspring during year two, the increased connectivity attributable to infant counts in year one, and the general assortativity patterns between other mothers – we cautiously extend that this effect could be a general social emergence, rather than a social preference for shared motherhood. That is, infants increase social associations, but as a general effect – not necessarily tied to present mothers. Grooming was much stronger during pregnancy, yet we found continuous increases in proximity and contact that peaked around birth. Thus, it is alternatively possible that pregnant females were engaging in preparatory social action. Under this interpretation, pregnant mothers would be engaging in social behavior that improves their social connectivity prior to postparturation when active socialization (e.g., grooming) is lower.

We found poor support for the premise that primiparous mothers invested more in triadic closure or had altered patterns of assortativity, relative to multiparous mothers. Despotic rhesus macaques often uphold normative dominance rank and kinship structures (Thierry, 2007). Given this matrilocal structure, what is the necessity of primiparous mothers to incorporate themselves in their group? Are there even opportunities for new connectivity that violates normative structures in a stable group? As of 2023, groups A and B had been established for 15 (though had experienced a social overthrow 9 years prior) and 6 years, respectively. Thus, the groups might be defined by long tenured relationships, akin to well established structural features and actions that uphold social norms (Wanat, 2010). Though there are still opportunities for flexible strategies that could alter clear unidirectional outcomes (Ellis et al., 2019), as evident by the cumulative effect of offspring count. Thus, maternal experience does matter when it comes in social behavior yet its role is one of continuous age-related change rather than a simple milestone of primiparity.

### 4.3. Variation in biomarker expression

We did not find strong evidence for improved physiological condition among females that had infants or juveniles and, thus, experienced shared motherhood. That is, mothers who had recently given birth or had an infant did not differ in their biomarkers relative to other females. We cannot fully distinguish between individuals that retain homeostasis due to a lack of challenges versus attain homeostasis through social support in the face of challenges. Even so, we found no evidence that social behavior was associated with cytokines or blood chemistry scores which weakens support for the action of social buffering against allostatic load. Alternatively, birth or infancy does not present a sufficient physiological challenge to alter cytokine or blood chemistry biomarkers.

This interpretation that infancy does not present a sufficient physiological challenge might be counterintuitive given the costs of lactation. The true costs of lactation are difficult to estimate, yet large-bodied primates are posited to increase their energetic needs by 30 % during lactation (Emery Thompson, 2024). Meeting this need is possible through several non-exclusive pathways: relying on body stores, adjusting foraging, increased feeding, or reduced physical activity (Emery Thompson, 2024). Provisioned rhesus macaques exhibited heightened glucocorticoid concentrations when lactating, based on within-subject and between-subject comparisons (Hoffman et al., 2011; Hoffman and Maestripieri, 2012). Yet, in line with our findings, cytokine concentrations (IL-1ra, IL-6, IL-8) did not differ between these reproductive stages despite an association between IL-8 and fecal glucocorticoid concentrations (Hoffman et al., 2011). Glucocorticoids and cytokines, while both implicated in allostatic load, have a complex interplay (Sapolsky et al., 2000). Though our study did not include female feeding or activity budgets, we note that lactating females did reduce active socialization with other adults (grooming) while exhibiting increased or sustained passive associations (contact-sitting, proximity). Future work would benefit from examining the energetics of these dynamics.

Our strongest predictors for biomarker factors were pregnancy relative to other reproductive statuses and breeding relative to the birth season. Both conditions resulted in lower scores on factors one, two, and four, yet higher scores on factor three. For pregnancy, lower blood chemistry measures have been related to increased plasma or hemodilution (Bakker et al., 2023; Chandra et al., 2012; Ibáñez-Contreras et al., 2013; Whittaker and Lind, 1993), though such an effect cannot account for all changes (Whittaker and Lind, 1993). Even so, the majority of our blood chemistry measures (15/23) were lower among pregnant females (Supplementary Figure 9).

Cytokines, however, were higher during these same periods; which, for our selected measures, is expected during a normal pregnancy – at least in humans and mice (Meyyazhagan et al., 2023; Orsi et al., 2006). Yet, self-reported stress during pregnancy has been shown to be associated with IL-6 from serum and stimulated lymphocytes (Coussons-Read et al., 2007). Thus, variation in cytokine production across pregnancy is still relevant, but likely more nuanced than the current approach can further develop upon.

During the breeding season, subjects on birth control had higher blood chemistry factor scores, yet similar cytokine scores, relative to non-mothers. This observation is logical given non-mothers would be expected to be cycling during the breeding season and blood loss during menses has been attributed to lower biomarker values in macaques – for calcium, albumin, total protein, and γ-glutamyl transferase (Perigard et al., 2016).

### 4.4. Management implications

Our findings have implications for the social dynamics of managed primate populations. In long-term groups, changes in reproductive status might be expected to inadvertently scale-up to alter intragroup dynamics. We have shown that the administration of birth control has social outcomes that alter group dynamics during breeding (via altered behavior and physiology) and birthing seasons (via the unusual state of not having an infant during the typical birth season). Management to reduce population density will alter the social dynamics of the group, an unanticipated yet important outcome with uncertain implications for long-term care. Furthermore, while cross-fostering or nursery rearing has shown a multitude of effects on infants (Sackett et al., 2006), we emphasize that the donor’s maternal experience – and that of her group members – is also dramatically altered. If we assume that maintaining stability is important for group welfare (Beisner et al., 2015; Flack et al., 2006, 2005; Oates-O’Brien et al., 2010; Pritchard et al., 2024), then unanticipated social outcomes are undesirable or difficult to accommodate from a management perspective. Thus, we solicit long-term investigations to understand the applied consequences of reproductive manipulations for group social dynamics in managed captive and free-ranging populations.

### 4.5. Limitations

We note that there are several inevitable limitations to our work. While our subject count was high, we were limited in the number of physiological samples that we could obtain. This reality is difficult to circumvent in socially-housed animals without extensive training. A subset of our cytokines exhibited high CVs. Even so, analyses that excluded these high CV samples did not exhibit a change in outcomes providing evidence that there was no clear consequence for mean-level differences. Finally, in this population female age and offspring count were tightly coupled, which is unsurprising given a high majority of group females typically give birth every year. Consequently, age and offspring count would be statistically indistinguishable without a much larger sample size.

## 5. Conclusions

Individuals embedded within a group both influence and are influenced by their social circumstances (Cote et al., 2011; Kannan et al., 2025; McCowan et al., 2011; Pritchard et al., 2024, 2023). Here we provide associative evidence that both of these circumstances are likely to be true for mothers, whereby maternal effects operate at the level of the individual (reproductive status) and at the scale of the group (progressive increases associated with bioavailability of infants). Of course, these dynamics are coupled. Reproductive status also influenced biomarkers, in the form of blood chemistry and cytokines, yet individual behavioral patterns were not associated with variation in biomarkers factor scores. Thus, we found no evidence that new mothers improved their social condition with a benefit towards maintaining homeostasis. These findings are important to further understand the maternal experience in socially complex groups, and the physiological costs associated with that experience.

## Supplementary Material

Supplementary Materials

## Figures and Tables

**Table 1:** Biomarker factor model, with salient loadings (≥ 0.40) in bold. Communality and uniqueness are represented by the column headers of *h*^*2*^ and *u*^2^, respectively.

Biomarker	Factor *System*				*h^2^*	*u^2^*	Complexity
	1 *Blood Protein*	2 *Tissue Damage*	3 *Inflammation*	4 *Liver Function*			
Total Protein	**0.87**	0.01	0.06	−0.21	0.68	0.32	1.1
Albumin	**0.87**	−0.02	0.00	0.13	0.84	0.16	1.0
Calcium	**0.80**	0.02	−0.01	0.07	0.70	0.30	1.0
Cholesterol	**0.59**	−0.01	−0.11	0.08	0.41	0.59	1.1
Sodium	**0.49**	0.11	−0.14	0.20	0.44	0.57	1.6
AST	0.00	**0.94**	0.00	0.09	0.91	0.09	1.0
CPK	−0.07	**0.75**	0.04	−0.05	0.52	0.48	1.0
LDH	0.08	**0.70**	−0.03	−0.14	0.52	0.49	1.1
IL-10	0.06	0.04	**0.78**	0.00	0.60	0.40	1.0
IL-6	−0.01	−0.04	**0.76**	0.00	0.58	0.42	1.0
TNF	−0.05	0.02	**0.69**	0.05	0.49	0.51	1.0
GGT	−0.01	−0.05	0.11	**0.75**	0.56	0.44	1.1
ALKP	0.10	0.08	−0.08	**0.74**	0.64	0.36	1.1

## Data Availability

Data has been made available on the Dryad Data Repository: https://doi.org/10.5061/dryad.w6m905r1n

## Appendix A. Supporting information

Supplementary data associated with this article can be found in the online version at doi:10.1016/j.anireprosci.2026.108149.
