# Supplementary material for: The social circumstances of the maternal experience and its biobehavioral associations, in rhesus macaques (Macaca mulatta)
Source: Anim Reprod Sci. Author manuscript; Available in PMC 2026 Apr 9. (PMC13060029; doi:10.1016/j.anireprosci.2026.108149)
Supplement: Supplementary Materials [file NIHMS2156670-supplement-Supplementary_Materials.pdf]

Supplementary Materials: The Social Circumstances of the Maternal Experience and its Biobehavioral Associations, in Rhesus Macaques (*Macaca mulatta)*

Alexander J. Pritchard[1,2], Rosemary A. Blersch[1,2], Emily M. Dura[1,3], Christina M. Nord[1,2], Amy C. Nathman[1], Jessica J. Vandeleest[1,2], Brenda McCowan[1,2]

[1] Neuroscience and Behavior Unit, California National Primate Research Center, University of California Davis, Davis, CA, USA

[2] Department of Population Health & Reproduction, School of Veterinary Medicine, University of California Davis, Davis, CA, USA

[3] Behavioral Management, California National Primate Research Center, University of California Davis, Davis, CA, USA

Citation:

Alexander J. Pritchard, Rosemary A. Blersch, Emily M. Dura, Christina M. Nord, Amy C. Nathman, Jessica J. Vandeleest, Brenda McCowan. 2026. The social circumstances of the maternal experience and its biobehavioral associations, in rhesus macaques (*Macaca mulatta*). *Animal Reproduction Science*, 108149. https://doi.org/10.1016/j.anireprosci.2026.108149

Supplementary Text 1

Four of the blood chemistry measures were excluded due to low MSA values: K, CL, hsCRP, CO_2, and TBIL. Examination of communalities led to the exclusion of: DBIL, TRIG, GLU, PHOS, ALT, and AGAP. A five factor model was deemed appropriate with acceptable MSA estimates. This model, however, had BUN fully defining a factor. We excluded BUN and then CREA, because of its communality. Factor selection suggested a four factor model. TNFα loaded highly on our cytokine factor, but was dominated by a large number of zero values. Thus, we recoded TNFα to a dichotomous variable and then ran a factor analysis using the weighted least squares factor method. This outcome was the final reported factor model in the manuscript.

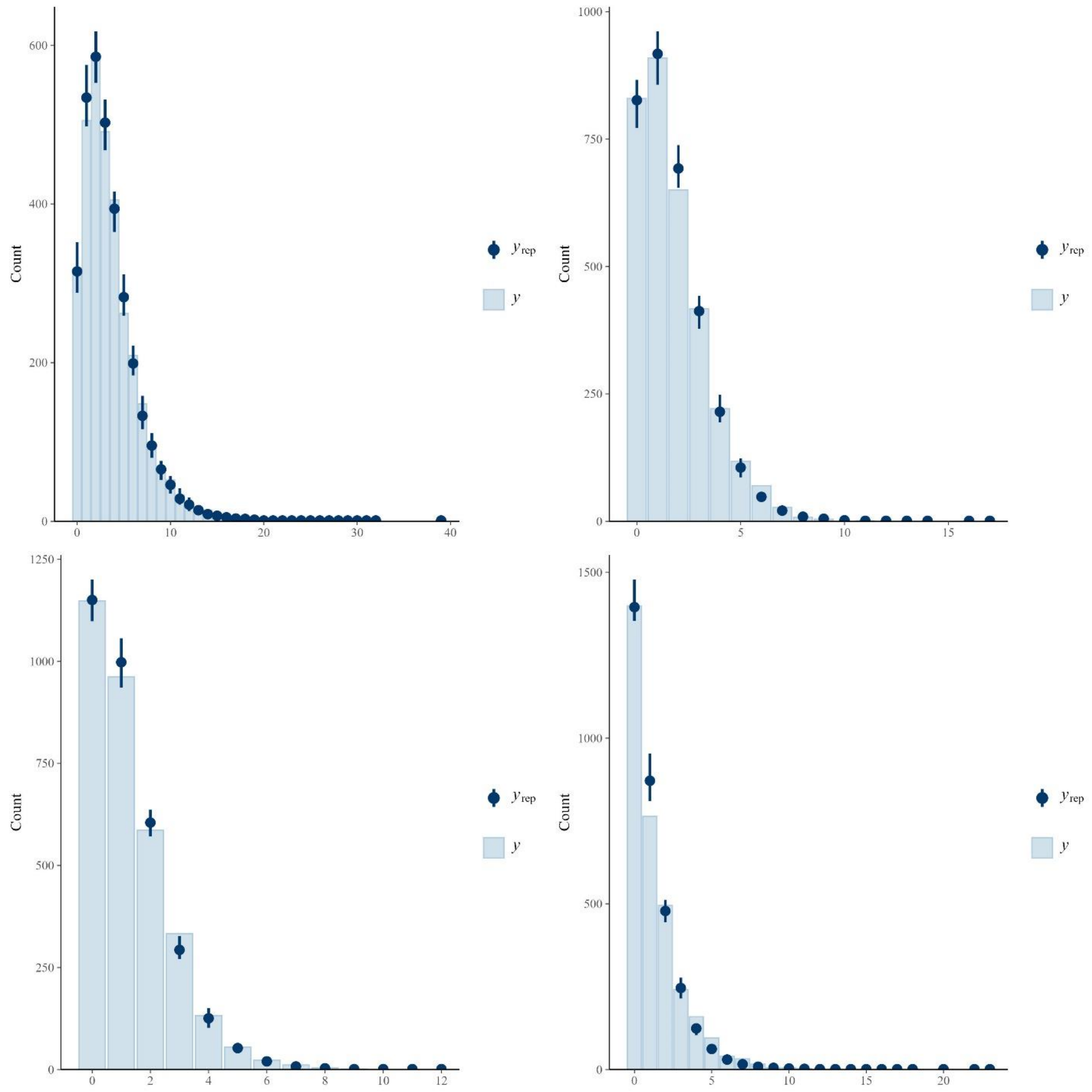

Supplementary Figure 1. Posterior predictive check bar plots for our behavioral models, comparing our known dataset (blue bars) versus the posteriors (dark blue point-and-whiskers). Each plot is for each of our response variables, clockwise from the top-left: Proximity, Grooming, Huddling, Contact-Sit.

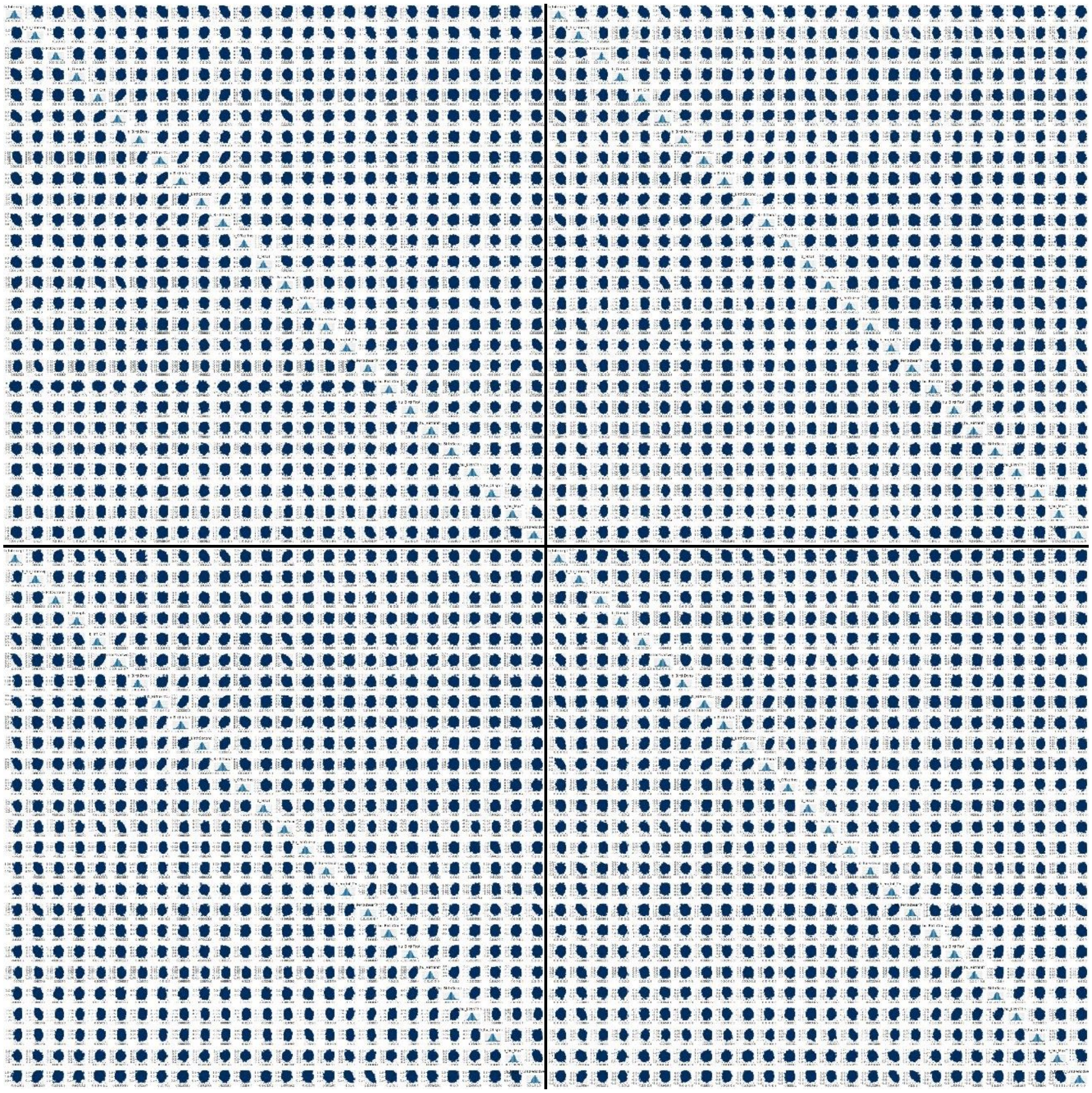

Supplementary Figure 2. Posterior pairs plots for our behavioral models. Each plot is for each of our response variables, clockwise from the top-left: Proximity, Grooming, Huddling, Contact-Sit.

Supplementary Table 1. Proximity univariate model posterior estimates, errors, credible intervals, and goodness-of-fit measures. Variables that credibly differ from zero are italicized

	Estimate	Error	l-95% CI	u-95% CI	$\hat{R}$ Estimate	Bulk ESS	Tail ESS
**Random Effects**							
**sd(ID)**	0.25	0.02	0.21	0.30	1	1568	2314
**sd(hu_ID)**	0.61	0.11	0.39	0.84	1	1542	2165
**Fixed Effects**							
Negative Binomial							
**Intercept**	0.70	0.06	0.58	0.82	1	1889	2303
**Rank**	0.32	0.06	0.21	0.43	1	1695	2511
GroupB	0.10	0.05	-0.01	0.21	1	1761	1998
**Inf_Cnt**	0.52	0.06	0.40	0.64	1	2590	2762
**Period_Year2**	0.18	0.04	0.10	0.27	1	2629	2657
BirthDonor	0.06	0.08	-0.10	0.21	1	2394	2627
**BirthFirst50**	0.39	0.04	0.30	0.47	1	2247	2474
**BirthInfant**	0.30	0.05	0.20	0.40	1	2374	2669
BirthSecondTri	-0.04	0.08	-0.20	0.12	1	2448	2561
**BirthThirdTri**	0.14	0.05	0.04	0.24	1	2140	2257
**Offspring**	0.15	0.05	0.05	0.26	1	1676	2240
**MaxT**	0.20	0.04	0.13	0.27	1	2773	2350
**Inf_Cnt:Period2**	-0.69	0.08	-0.84	-0.54	1	2471	2522
Hurdle							
**hu_Intercept**	-1.27	0.19	-1.64	-0.91	1	2429	2551
**Rank**	-0.78	0.20	-1.20	-0.39	1	2348	2493
GroupB	-0.05	0.18	-0.41	0.31	1	2395	2536
**Inf_Cnt**	-1.46	0.24	-1.91	-0.99	1	2711	2653
**Period2**	-0.47	0.17	-0.81	-0.14	1	2681	2749
BirthDonor	-0.66	0.36	-1.38	0.01	1	2805	2677
**BirthFirst50**	-1.51	0.25	-2.02	-1.03	1	2524	2479
**BirthInfant**	-1.24	0.32	-1.91	-0.65	1	2689	2545
**BirthSecondTri**	-1.06	0.33	-1.75	-0.43	1	2500	2726
**BirthThirdTri**	-1.09	0.22	-1.54	-0.67	1	2467	2407
**Offspring**	-0.55	0.21	-0.96	-0.14	1	2667	2675
**MaxT**	-0.43	0.19	-0.80	-0.08	1	2713	2583
**Inf_Cnt:Period2**	1.77	0.35	1.07	2.42	1	2820	2726
**Distribution**							
**Shape**	5.90	0.51	4.99	6.99	1	2765	2287

Supplementary Table 2. Contact-sit univariate model posterior estimates, errors, credible intervals, and goodness-of-fit measures. Variables that credibly differ from zero are italicized

	Estimate	Error	l-95% CI	u-95% CI	$\hat{R}$ Estimate	Bulk ESS	Tail ESS
**Random Effects**							
**sd(ID)**	0.23	0.03	0.17	0.30	1	2053	2471
**sd(hu_ID)**	0.43	0.06	0.32	0.56	1	1808	2312
**Fixed Effects**							
Negative Binomial							
Intercept	-0.11	0.09	-0.30	0.06	1	2462	2868
Rank	0.11	0.07	-0.03	0.24	1	2614	2786
**GroupB**	-0.38	0.07	-0.51	-0.25	1	2647	2531
**Inf_Cnt**	0.69	0.11	0.46	0.91	1	2528	2796
**Period_Year2**	0.42	0.07	0.28	0.57	1	2640	2584
**BirthDonor**	0.26	0.13	0.00	0.52	1	2427	2660
**BirthFirst50**	0.29	0.07	0.15	0.41	1	2446	2627
**BirthInfant**	0.27	0.08	0.11	0.43	1	2727	2780
BirthSecondTri	0.19	0.12	-0.06	0.42	1	2560	2642
**BirthThirdTri**	0.30	0.08	0.15	0.44	1	2393	2655
**Offspring**	0.21	0.06	0.09	0.34	1	2390	2546
MaxT	-0.10	0.06	-0.22	0.03	1	2791	2725
**Inf_Cnt:Period2**	-0.75	0.13	-1.01	-0.50	1	2673	2618
Hurdle							
hu_Intercept	0.14	0.13	-0.12	0.40	1	2671	2508
**Rank**	-0.29	0.13	-0.54	-0.05	1	2601	2625
**GroupB**	0.72	0.12	0.48	0.96	1	2717	2718
**Inf_Cnt**	-1.33	0.16	-1.65	-1.01	1	2337	2319
**Period2**	-0.86	0.11	-1.07	-0.63	1	2659	2700
BirthDonor	-0.15	0.21	-0.57	0.28	1	2539	2686
**BirthFirst50**	-0.67	0.13	-0.92	-0.44	1	2448	2797
**BirthInfant**	-0.54	0.15	-0.86	-0.25	1	2260	2361
BirthSecondTri	-0.37	0.20	-0.76	0.04	1	2608	2520
**BirthThirdTri**	-0.79	0.14	-1.05	-0.51	1	2408	2435
**Offspring**	-0.26	0.12	-0.49	-0.03	1	2575	2691
MaxT	0.05	0.11	-0.16	0.26	1	2514	2544
**Inf_Cnt:Period2**	1.08	0.22	0.67	1.51	1	2837	2483
**Distribution**							
**Shape**	17.65	17.17	6.73	58.20	1	2101	2427

Supplementary Table 3. Grooming univariate model posterior estimates, errors, credible intervals, and goodness-of-fit measures. Variables that credibly differ from zero are italicized

	Estimate	Error	l-95% CI	u-95% CI	$\hat{R}$ Estimate	Bulk ESS	Tail ESS
**Random Effects**							
**sd(ID)**	0.24	0.03	0.18	0.30	1	1880	2483
**sd(hu_ ID)**	0.45	0.07	0.32	0.58	1	2011	2433
**Fixed Effects**							
Negative Binomial							
**Intercept**	0.35	0.07	0.21	0.48	1	2171	2659
**Rank**	0.41	0.06	0.28	0.53	1	2486	2558
GroupB	-0.09	0.06	-0.21	0.02	1	2485	2486
**Inf_Cnt**	0.37	0.08	0.22	0.52	1	2778	2874
**Period_Year2**	0.19	0.05	0.09	0.30	1	2658	2711
BirthDonor	-0.02	0.12	-0.25	0.20	1	2753	2892
**BirthFirst50**	-0.12	0.06	-0.24	-0.01	1	2454	2509
**BirthInfant**	-0.16	0.07	-0.31	-0.02	1	2506	2647
**BirthSecondTri**	0.21	0.09	0.03	0.38	1	2015	2185
**BirthThirdTri**	0.26	0.06	0.14	0.37	1	2209	2265
**Offspring**	0.15	0.06	0.04	0.27	1	2298	2484
**MaxT**	0.11	0.05	0.01	0.21	1	2765	2686
**Inf_Cnt:Period2**	-0.70	0.10	-0.89	-0.51	1	2534	2665
Hurdle							
**hu_Intercept**	-0.73	0.14	-1.01	-0.46	1	2700	2494
**Rank**	-0.60	0.13	-0.87	-0.35	1	2375	2326
GroupB	0.10	0.12	-0.14	0.33	1	2618	2560
**Inf_Cnt**	-0.65	0.18	-0.98	-0.31	1	2868	2593
**Period2**	-0.30	0.12	-0.54	-0.05	1	2506	2724
BirthDonor	0.19	0.23	-0.27	0.62	1	2036	2450
BirthFirst50	0.18	0.13	-0.08	0.43	1	2331	2792
BirthInfant	0.26	0.16	-0.07	0.57	1	2670	2766
**BirthSecondTri**	-0.72	0.25	-1.20	-0.22	1	2482	2578
**BirthThirdTri**	-0.74	0.15	-1.05	-0.45	1	2573	2762
**Offspring**	-0.38	0.13	-0.64	-0.12	1	2755	2762
**MaxT**	-0.53	0.12	-0.75	-0.30	1	2767	2432
**Inf_Cnt:Period2**	1.32	0.24	0.86	1.78	1	2419	2513
**Distribution**							
**Shape**	12.76	5.04	7.24	25.41	1	2557	2410

Supplementary Table 4. Huddling univariate model posterior estimates, errors, credible intervals, and goodness-of-fit measures. Variables that credibly differ from zero are italicized

	Estimate	Error	l-95% CI	u-95% CI	$\hat{R}$ Estimate	Bulk ESS	Tail ESS
**Random Effects**							
**sd(ID)**	0.26	0.04	0.18	0.34	1	1427	2424
**sd(hu_ID)**	0.56	0.06	0.44	0.69	1	1866	2389
**Fixed Effects**							
Negative Binomial							
**Intercept**	0.51	0.09	0.33	0.67	1	2703	2690
Rank	-0.07	0.08	-0.22	0.08	1	2619	2387
GroupB	-0.06	0.07	-0.21	0.08	1	2776	2544
Inf_Cnt	0.07	0.10	-0.11	0.26	1	2748	2910
**Period_Year2**	-0.46	0.07	-0.59	-0.33	1	2736	2504
BirthDonor	0.15	0.18	-0.20	0.49	1	2547	2384
**BirthFirst50**	0.31	0.08	0.16	0.47	1	2184	2549
**BirthInfant**	0.33	0.11	0.11	0.55	1	2471	2656
BirthSecondTri	0.18	0.13	-0.08	0.45	1	2703	2613
**BirthThirdTri**	0.48	0.08	0.32	0.65	1	2554	2560
Offspring	-0.12	0.08	-0.27	0.03	1	2861	2560
**MaxT**	-0.67	0.07	-0.81	-0.54	1	2747	2798
Inf_Cnt:Period2	0.21	0.15	-0.09	0.50	1	2802	2740
Hurdle							
**hu_Intercept**	-0.84	0.14	-1.12	-0.57	1	2762	2548
Rank	0.18	0.14	-0.10	0.45	1	2568	2726
GroupB	0.10	0.13	-0.16	0.37	1	2600	2814
Inf_Cnt	-0.32	0.17	-0.64	0.02	1	2814	2799
**Period2**	1.16	0.12	0.93	1.39	1	2712	2624
**BirthDonor**	-0.57	0.22	-1.00	-0.14	1	2557	2638
**BirthFirst50**	-0.62	0.13	-0.87	-0.38	1	2774	2637
BirthInfant	0.18	0.15	-0.12	0.49	1	2608	2390
**BirthSecondTri**	-0.71	0.21	-1.14	-0.31	1	2652	2693
**BirthThirdTri**	-1.13	0.14	-1.42	-0.86	1	2653	2618
Offspring	0.17	0.14	-0.10	0.43	1	2401	2523
**MaxT**	1.26	0.12	1.04	1.49	1	2591	2511
Inf_Cnt:Period2	-0.37	0.22	-0.79	0.06	1	2578	2541
**Distribution**							
**Shape**	2.67	0.43	1.95	3.64	1	2380	2576

Supplementary Table 5. Bayesian R^2 estimates for our behavioral models.

			Quantiles	
**R^2 Estimate**	**Estimate**	**Error**	**2.5%**	**97.5%**
		**Proximity**		
Conditional	0.33	0.02	0.30	0.36
Marginal	0.23	0.02	0.18	0.27
		**Contact-Sit**		
Conditional	0.16	0.01	0.14	0.19
Marginal	0.12	0.01	0.09	0.14
		**Grooming**		
Conditional	0.19	0.01	0.17	0.22
Marginal	0.13	0.02	0.10	0.16
		**Huddling**		
Conditional	0.21	0.02	0.18	0.24
Marginal	0.16	0.01	0.13	0.19

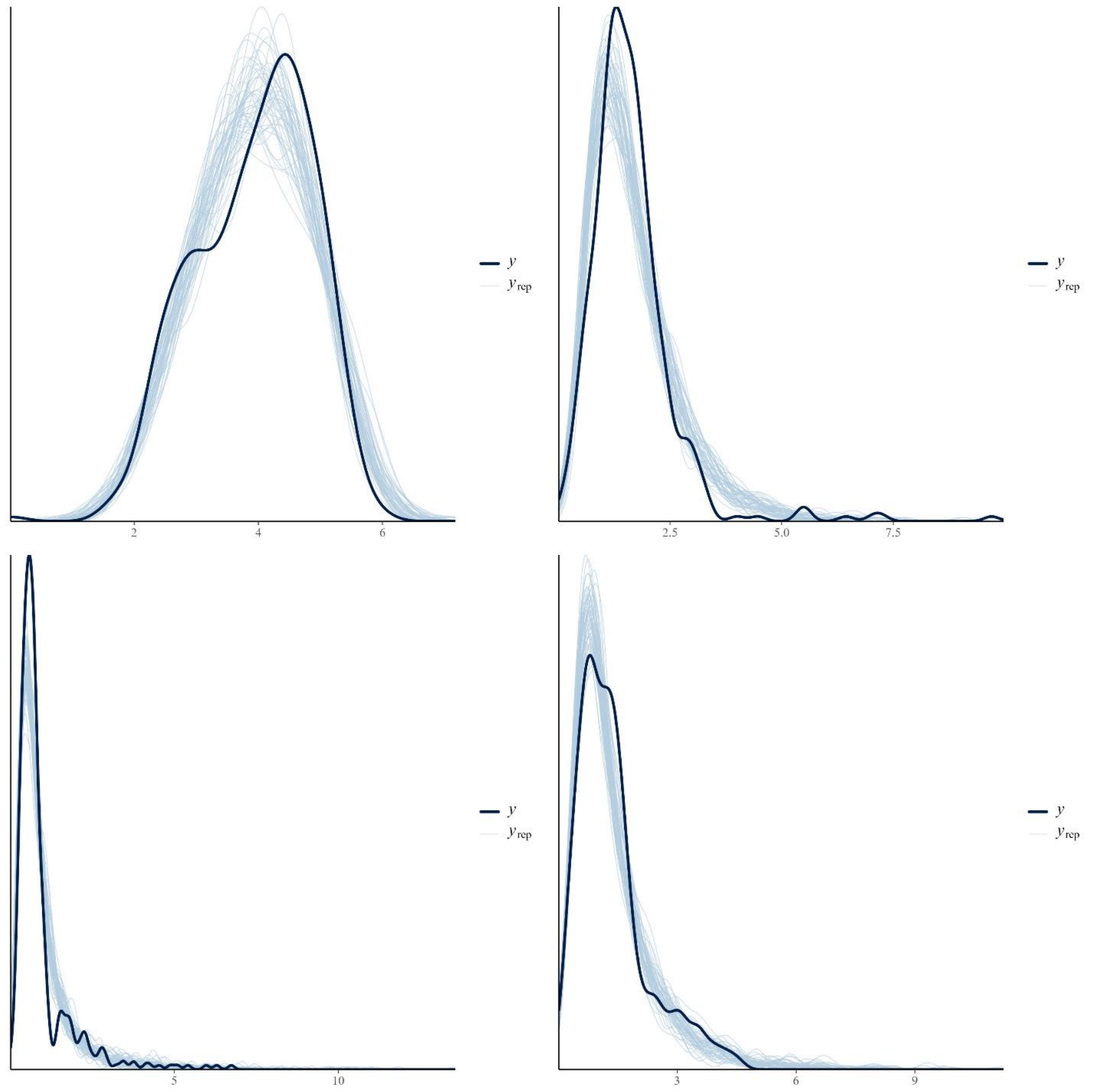

Supplementary Figure 3. Posterior predictive check density plots for our biomarker models, comparing our known dataset (dark blue density) versus the posteriors (blue traces [N = 50]). Each plot is for each of our response variables, clockwise from the top-left: Factor One, Factor Two, Factor Four, Factor Three.

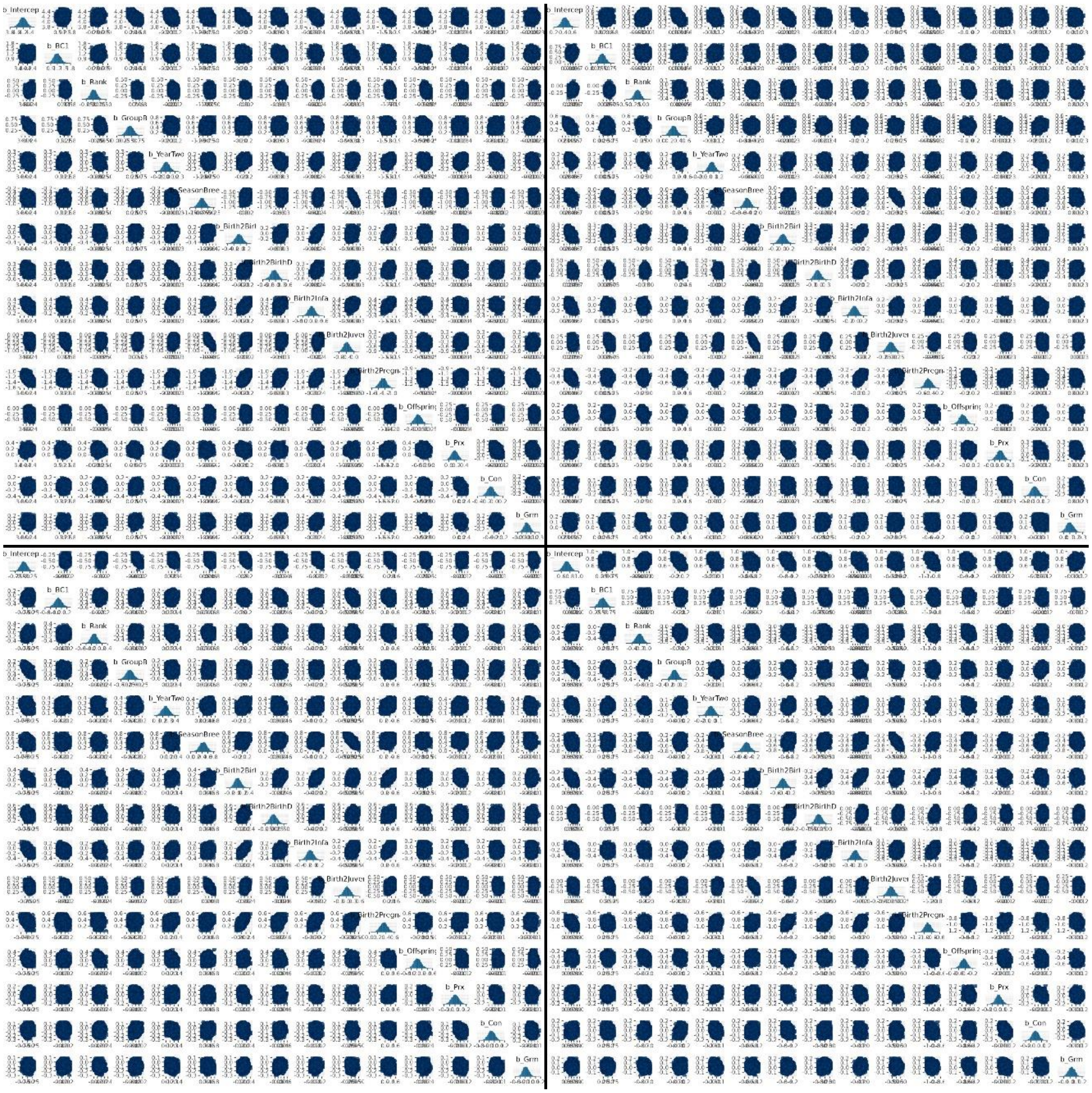

Supplementary Figure 4. Pairs plots for our biomarker models. Each plot is for each of our response variables, clockwise from the top-left: Factor One, Factor Two, Factor Four, Factor Three.

Supplementary Table 6. Factor one univariate model posterior estimates, errors, credible intervals, and goodness-of-fit measures. Variables that credibly differ from zero are italicized

	Estimate	Error	l-95% CI	u-95% CI	$\hat{R}$ Estimate	Bulk ESS	Tail ESS
**Random Effects**							
**sd(ID)**	0.50	0.05	0.4	0.61	1	2373	3329
**Fixed Effects**							
**Intercept**	4.19	0.11	3.98	4.40	1	2744	3799
**On Birth Control**	1.19	0.15	0.89	1.50	1	3918	4213
Rank	0.03	0.12	-0.21	0.26	1	2795	3866
**GroupB**	0.45	0.12	0.23	0.69	1	2511	3015
Year Two	0.01	0.07	-0.13	0.15	1	3953	4326
**Breeding**	-0.74	0.14	-1.02	-0.47	1	4481	4468
Birth	-0.04	0.11	-0.25	0.18	1	3221	4233
Donor	-0.07	0.17	-0.40	0.26	1	3370	4141
Infant	0.12	0.12	-0.10	0.35	1	3534	4191
**Juvenile**	-0.38	0.16	-0.68	-0.06	1	3885	4476
**Pregnancy**	-1.26	0.10	-1.46	-1.06	1	3082	4247
**Offspring**	-0.25	0.13	-0.50	-0.00	1	2499	3264
Proximity	0.15	0.08	-0.00	0.31	1	4554	4781
Contact-Sit	-0.09	0.08	-0.24	0.07	1	4358	4427
Grooming	0.01	0.07	-0.13	0.15	1	4311	4186
**Distribution**							
**Sigma**	0.50	0.02	0.47	0.55	1	3858	4457

Supplementary Table 7. Factor two univariate model posterior estimates, errors, credible intervals, and goodness-of-fit measures. Variables that credibly differ from zero are italicized

	Estimate	Error	l-95% CI	u-95% CI	$\hat{R}$ Estimate	Bulk ESS	Tail ESS
**Random Effects**							
**sd(ID)**	0.27	0.03	0.22	0.34	1	2616	4071
**Fixed Effects**							
**Intercept**	0.44	0.07	0.30	0.58	1	3595	4017
**On Birth Control**	0.44	0.12	0.21	0.68	1	4044	4242
Rank	-0.15	0.08	-0.31	0.00	1	4342	4707
**GroupB**	0.29	0.07	0.15	0.44	1	3403	4464
Year Two	0.07	0.05	-0.04	0.17	1	4214	4142
**Breeding**	-0.38	0.11	-0.60	-0.17	1	4331	4502
Birth	0.02	0.08	-0.14	0.18	1	4062	4241
Donor	-0.05	0.13	-0.29	0.20	1	3987	4063
Infant	-0.04	0.09	-0.20	0.13	1	3974	3975
Juvenile	-0.01	0.12	-0.25	0.23	1	4252	4644
**Pregnancy**	-0.44	0.08	-0.59	-0.29	1	3762	4215
Offspring	-0.07	0.08	-0.22	0.09	1	3923	4605
Proximity	0.10	0.06	-0.02	0.23	1	4574	4910
Contact-Sit	-0.01	0.06	-0.14	0.11	1	4488	4805
Grooming	0.10	0.05	-0.01	0.20	1	4244	4223
**Distribution**							
**Sigma**	6.28	0.50	5.35	7.33	1	3675	4089

Supplementary Table 8. Factor three univariate model posterior estimates, errors, credible intervals, and goodness-of-fit measures. Variables that credibly differ from zero are italicized

	Estimate	Error	l-95% CI	u-95% CI	$\hat{R}$ Estimate	Bulk ESS	Tail ESS
**Random Effects**							
**sd(ID)**	0.56	0.05	0.47	0.67	1	1813	3152
**Fixed Effects**							
**Intercept**	-0.46	0.11	-0.66	-0.25	1	1803	3060
On Birth Control	-0.12	0.13	-0.37	0.14	1	4126	4499
Rank	-0.10	0.11	-0.32	0.12	1	2304	3297
GroupB	-0.20	0.13	-0.45	0.05	1	1619	2810
**Year Two**	0.25	0.06	0.13	0.36	1	3271	3991
**Breeding**	0.44	0.12	0.21	0.67	1	4453	4618
Birth	0.04	0.09	-0.14	0.22	1	2900	4119
Donor	0.08	0.14	-0.19	0.36	1	3481	4101
Infant	-0.09	0.10	-0.29	0.10	1	3050	3857
Juvenile	0.04	0.14	-0.23	0.31	1	3751	4170
**Pregnancy**	0.35	0.09	0.18	0.52	1	2619	3836
Offspring	0.03	0.13	-0.22	0.28	1	1557	2759
Proximity	0.00	0.07	-0.13	0.13	1	4777	4559
Contact-Sit	-0.06	0.07	-0.19	0.07	1	3893	4291
Grooming	-0.09	0.06	-0.21	0.02	1	4327	4286
**Distribution**							
**Sigma**	6.01	0.48	5.09	6.99	1	3759	4291

Supplementary Table 9. Factor four univariate model posterior estimates, errors, credible intervals, and goodness-of-fit measures. Variables that credibly differ from zero are italicized

	Estimate	Error	l-95% CI	u-95% CI	$\hat{R}$ Estimate	Bulk ESS	Tail ESS
**Random Effects**							
**sd(ID)**	0.34	0.04	0.28	0.42	1	2667	3925
**Fixed Effects**							
**Intercept**	0.73	0.08	0.57	0.87	1	3049	3751
**On Birth Control**	0.50	0.11	0.28	0.72	1	3982	4147
**Rank**	-0.20	0.08	-0.36	-0.03	1	3244	3880
GroupB	-0.04	0.08	-0.20	0.13	1	2918	3630
Year Two	-0.02	0.05	-0.12	0.08	1	3967	4237
**Breeding**	-0.45	0.11	-0.66	-0.23	1	4425	4534
**Birth**	-0.40	0.08	-0.56	-0.24	1	3234	4167
**Donor**	-0.29	0.12	-0.53	-0.04	1	3748	4318
**Infant**	-0.21	0.09	-0.38	-0.04	1	3730	3969
**Juvenile**	-0.24	0.12	-0.49	-0.01	1	3957	4078
**Pregnancy**	-0.90	0.08	-1.05	-0.75	1	3133	3914
**Offspring**	-0.45	0.09	-0.62	-0.28	1	3101	3723
Proximity	0.02	0.06	-0.10	0.14	1	4334	4028
Contact-Sit	-0.01	0.06	-0.13	0.11	1	4133	4452
Grooming	0.03	0.05	-0.08	0.13	1	4470	4409
**Distribution**							
**Sigma**	6.95	0.56	5.91	8.09	1	3639	4592

Supplementary Table 10. Bayesian R^2 estimates for our behavioral models.

			Quantiles	
**R^2 Estimate**	**Estimate**	**Error**	**2.5%**	**97.5%**
	**Factor One**			
Conditional	0.72	0.02	0.68	0.75
Marginal	0.49	0.03	0.44	0.54
	**Factor Two**			
Conditional	0.64	0.04	0.56	0.70
Marginal	0.06	0.02	0.03	0.11
	**Factor Three**			
Conditional	0.42	0.04	0.35	0.50
Marginal	0.20	0.03	0.14	0.27
	**Factor Four**			
Conditional	0.78	0.03	0.72	0.83
Marginal	0.54	0.06	0.42	0.64

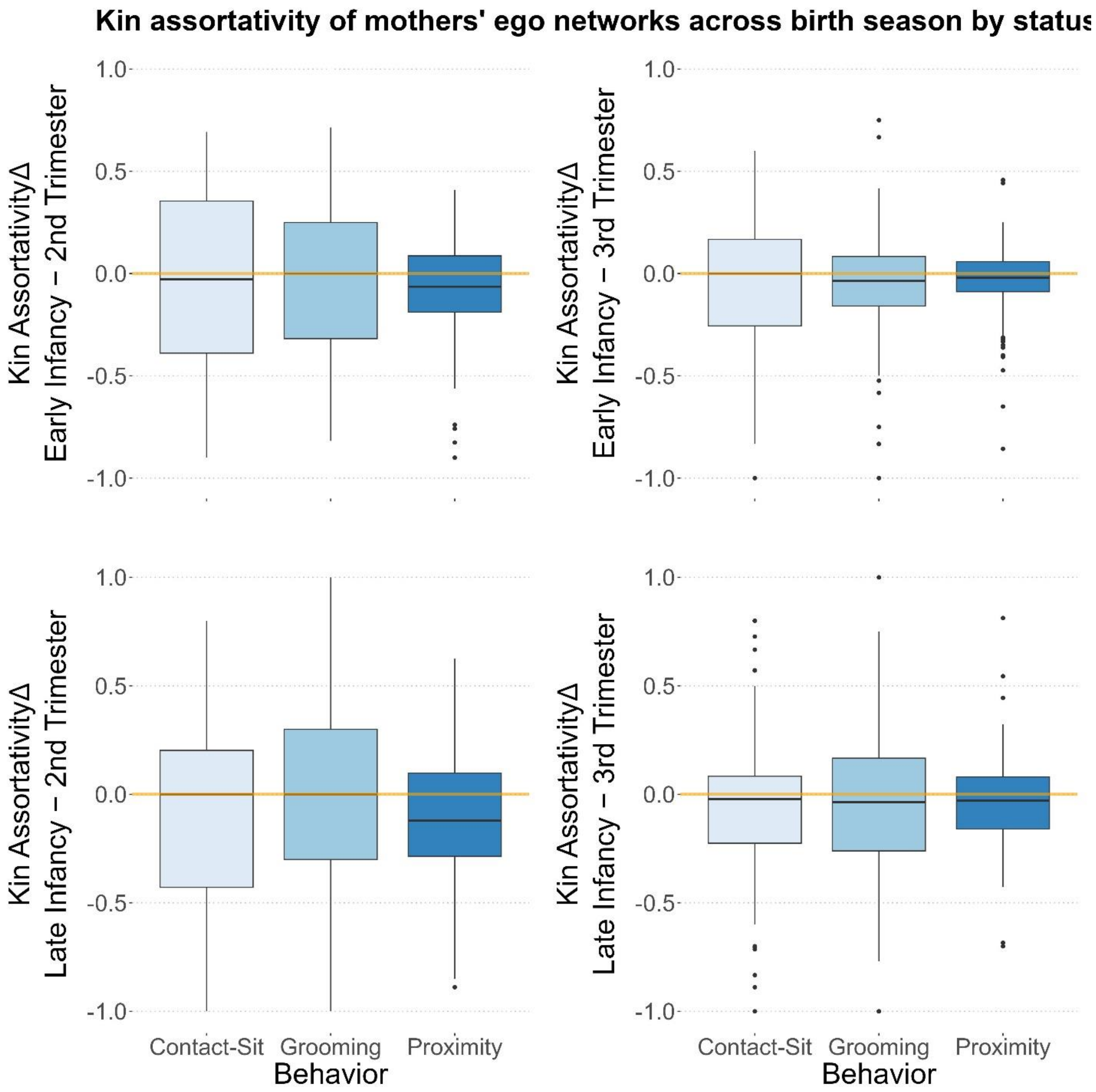

Supplementary Figure 5. Boxplots of changes to kin assortativity with early (top row) or late infancy (bottom row) minus assortativity indices from the second (left column) or third trimester (right column) for each of the four behavior (x-axis and fill color). Note that equivalence between two periods is emphasized by 0 at the y-intercept.

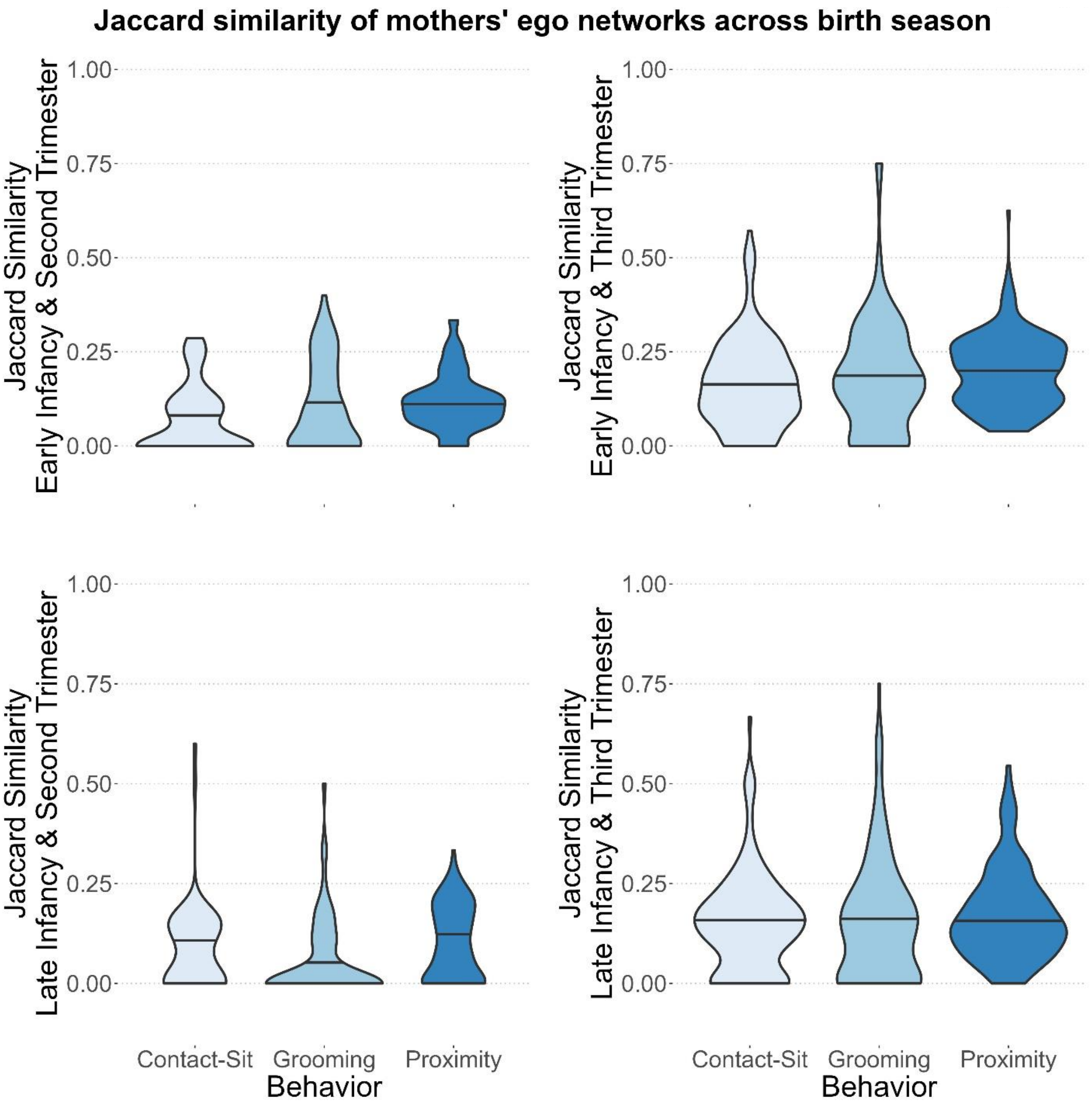

Supplementary Figure 6. Violin plot of Jaccard similarity indices (y-axes) for each of the behaviors (x-axes; fill color). Plots are organized with early (top row) or late infancy (bottom row) relative to the second (left column) or third trimester (right column).

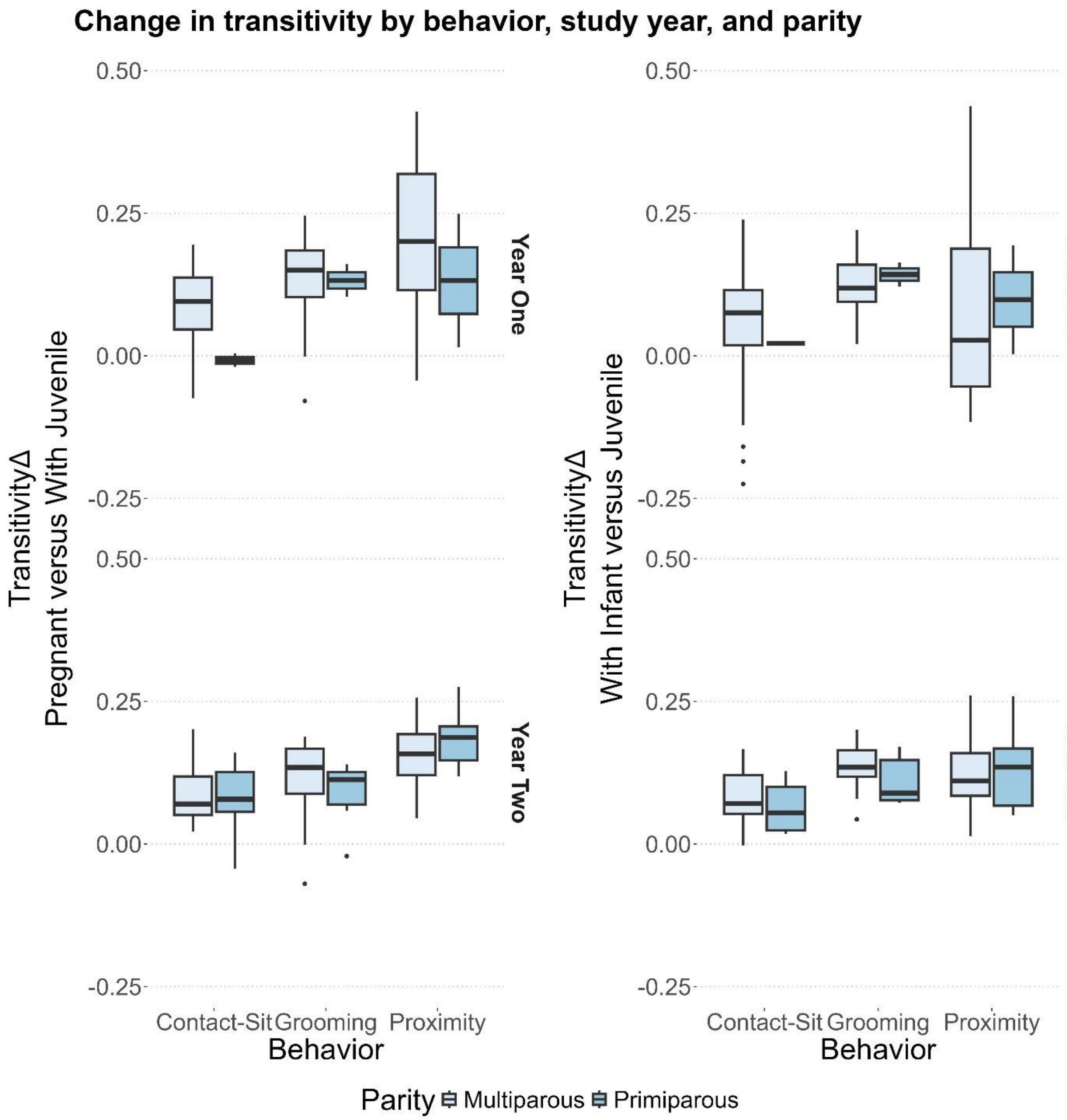

Supplementary Figure 7. Boxplots of the change in transitivity for second-neighbor ego networks. Change in transitivity (y-axes) for each of the behaviors (x-axes) for primiparous (darker boxes) and multiparous (lighter boxes). Plots are organized with year one (top row) or two (bottom row), and pregnancy versus juvenility (left column) or infancy versus juvenility (right column). The change in transitivity is the juvenility period minus non-juvenility, with higher scores indicating greater transitivity with juvenility.

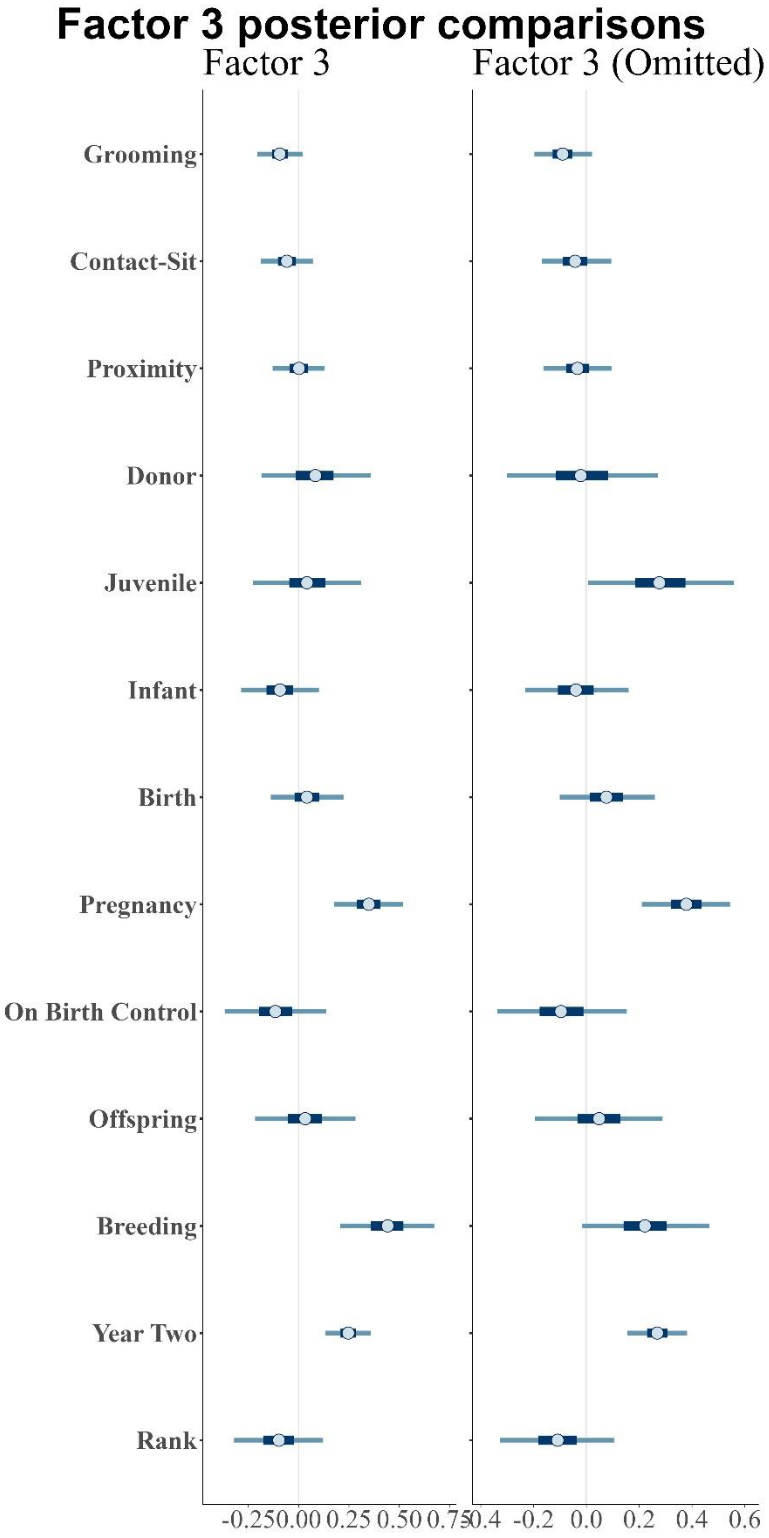

Supplementary Figure 8. Posterior estimates for the factor 3 univariate biomarker model with omitted samples (right panel), compared to the final model from the manuscript (left panel). Points show the median estimate, with inner intervals extending to the 50% credible interval, and outer intervals to the 95% credible interval. Modelled fixed effect variables are on the y-axis.

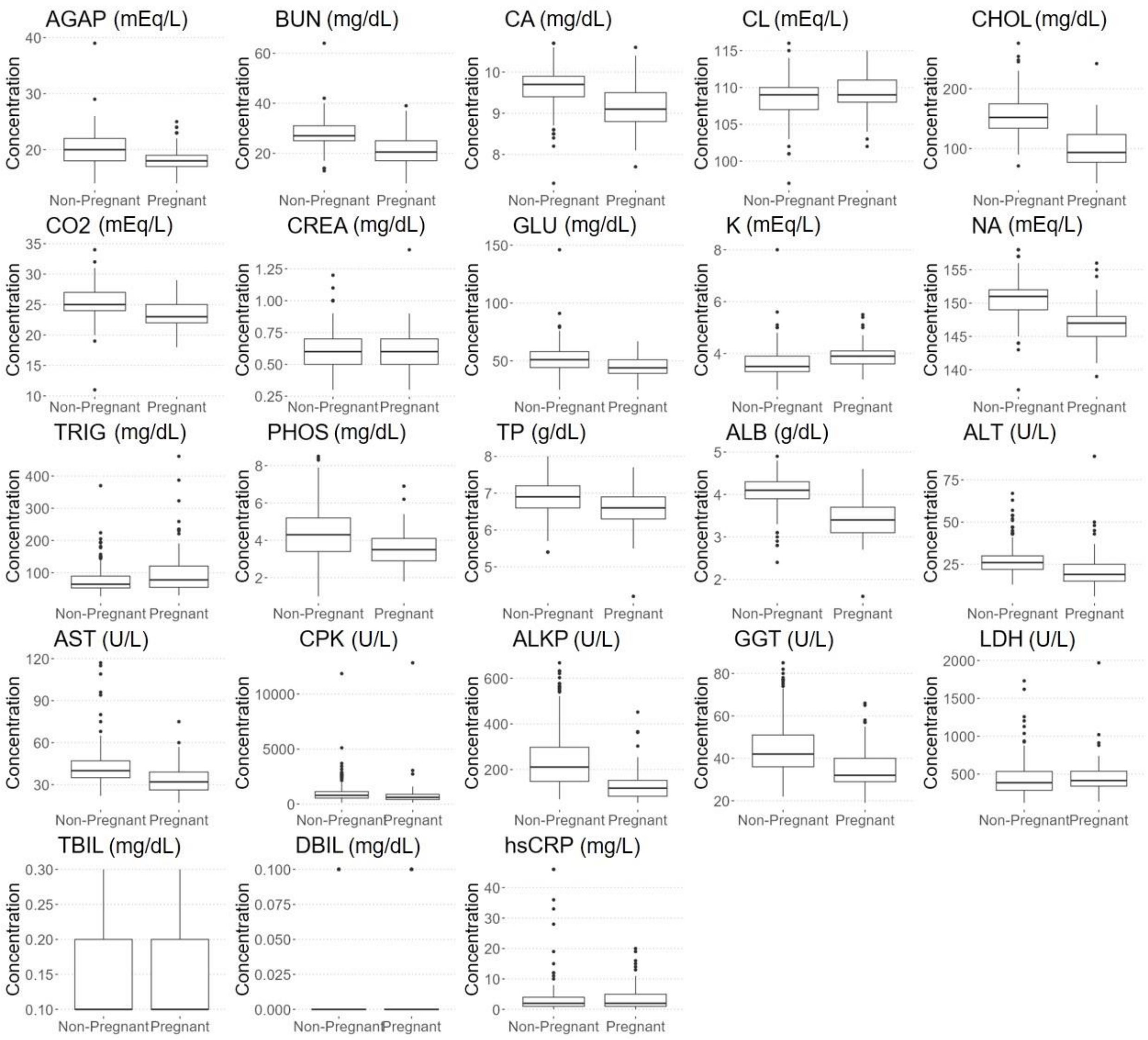

Supplementary Figure 9. Boxplots comparing blood chemistry values (y-axes) for all samples taken during majority non-pregnancy and majority pregnancy periods (x-axes). Plots are labelled in the top-left.
